# Developmental pathways to autism: A review of prospective studies of infants at risk^☆^

**DOI:** 10.1016/j.neubiorev.2013.12.001

**Published:** 2014-02

**Authors:** Emily J.H. Jones, Teodora Gliga, Rachael Bedford, Tony Charman, Mark H. Johnson

**Affiliations:** a Centre for Brain and Cognitive Development, Birkbeck College, University of London, UK; b King's College London, Institute of Psychiatry, Department of Psychology, UK; c King's College London, Institute of Psychiatry, Department of Biostatistics, UK

**Keywords:** ASD, Autism, Infant sibling, High-risk, Causal path, Developmental mechanisms

## Abstract

Autism Spectrum Disorders (ASDs) are neurodevelopmental disorders characterized by impairments in social interaction and communication, and the presence of restrictive and repetitive behaviors. Symptoms of ASD likely emerge from a complex interaction between pre-existing neurodevelopmental vulnerabilities and the child's environment, modified by compensatory skills and protective factors. Prospective studies of infants at high familial risk for ASD (who have an older sibling with a diagnosis) are beginning to characterize these developmental pathways to the emergence of clinical symptoms. Here, we review the range of behavioral and neurocognitive markers for later ASD that have been identified in high-risk infants in the first years of life. We discuss theoretical implications of emerging patterns, and identify key directions for future work, including potential resolutions to several methodological challenges for the field. Mapping how ASD unfolds from birth is critical to our understanding of the developmental mechanisms underlying this disorder. A more nuanced understanding of developmental pathways to ASD will help us not only to identify children who need early intervention, but also to improve the range of interventions available to them.

## Highlights

• Prospective studies of infants at familial risk are characterizing developmental pathways to ASD.
• Children with ASD show social and communication difficulties in the second year of life.
• Early neurocognitive markers include atypical neural response to gaze and slowed disengagement.
• Mapping how ASD unfolds from birth is central to early identification and intervention.

## 1 Introduction

Autism Spectrum Disorders (ASDs) are neurodevelopmental disorders characterized by impairments in social interaction and communication, and the presence of restrictive and repetitive behaviors (DSM-5, APA, 2013; ICD-10, WHO, 1993). In addition, there is significant comorbidity between ASD and clinically significant difficulties in a number of neurodevelopmental domains, including attention (e.g. Hanson et al., 2012), mood (e.g. Kim et al., 2000), cognitive skills (e.g. Charman et al., 2011a,b), and adaptive skills (e.g. Perry et al., 2009). One of the diagnostic features of ASD is its early emergence; symptoms must begin in early childhood for a diagnosis to be given. Detailed work with retrospective parent report and home-videos of young children with ASD has consistently shown that children who are later diagnosed with ASD show impairments in a range of skills in the first years of life (for review, Barbaro and Dissanayake, 2009; Yirmiya and Charman, 2010). As a developmental disorder, symptoms of ASD likely emerge from a complex interaction between pre-existing vulnerabilities and the child's environment. Initial genetic and environmental risk factors interact to alter the development of brain structure and function, compromising the child's ability to learn from their environment (Johnson, 2011). Early emerging behavioral symptoms alter the child's self-directed patterns of attention, changing their experience of the environment and further restricting social learning opportunities. Compensatory skills and pre-existing protective factors are also likely to play a role in the dynamics of a clinical phenotype. Understanding how ASD unfolds from birth onwards is critical to beginning to understand these developmental mechanisms, for identifying children who require early intervention and to indicate appropriate intervention targets.

Retrospective work is valuable but has many limitations; for example, memory or videotaped events may be selective, and researchers are limited to the assessment of overt behaviors that have been captured on tape or that are memorable to parents. To overcome these challenges, researchers have recently turned to prospective longitudinal studies of infants at high familial risk for ASD. Recent estimates suggested that ASD is moderately heritable (Hallmayer et al., 2011), with recurrence rates within families in community samples estimated to be around 10% (Constantino et al., 2010) compared to a population prevalence of ∼1% (Baird et al., 2006). Prospective studies of infants who later develop ASD are thus feasible within a familial high-risk design. Such studies of *high-risk infant siblings* follow younger siblings of children with the disorder from early infancy until 2–3 years of age, when a diagnosis of ASD can be made. A low-risk control group, composed of children with a typically developing older sibling and who have no family history of ASD, is typically followed in parallel. Around 20% of high-risk infant siblings meet criteria for ASD by their third birthday (Ozonoff et al., 2011); by comparing prospective data collected from infants who later do or do not meet diagnostic criteria for an ASD, researchers can identify early markers of later diagnosis. Of note, the lower sibling recurrence rate in community samples (c. 10%, Constantino et al., 2010) likely reflects a combination of “stoppage effects” (choosing not to have additional children if one child has a disability) and failure to detect milder forms of ASD in the community.

High-risk infant sibling designs also allow for the investigation of the broader autism phenotype (BAP, Bolton et al., 1994), subclinical traits or characteristics that are present at an elevated rate in family members of individuals with ASD. Around 10–20% of high-risk infants develop such sub-clinical ASD symptoms or other developmental problems (Messinger et al., 2013). Studying infants prospectively allows researchers to observe behavior in a more standardized context, and the use of a wider range of tools such as eye-tracking and neuroimaging allows inferences about underlying mechanisms. These rich datasets should enable both the development of new clinical screening tools for early behavioral signs of ASD, and new models of the developmental pathways leading to ASD and other related disorders.

Previous reviews in this area have identified several common themes (Elsabbagh and Johnson, 2010; Rogers, 2009; Yirmiya and Charman, 2010). Firstly, few behavioral markers have been identified in the first year of life. Rather, observable behavioral impairments appear to accumulate across the second year of life. Second, rather than observing clear early impairments in social behavior that precede impairments in other domains, early symptoms are apparent across multiple domains including sensory and repetitive behaviors as well as impairments in early social communicative behaviors. This review is motivated by the need to revisit and extend these conclusions based on the many subsequent studies that have emerged since the publication of these recent reviews.

We draw conclusions in two key areas. First, we consider the implications of reviewed findings for theoretical accounts of the development of ASD. For example, social orienting models of ASD suggest that an early emerging reduction in social attention compromises the child's opportunities to learn about their social environment, contributing to the development of symptoms of ASD. This account thus predicts that deficits in social orienting should emerge before other signs of ASD. Prospective work with infant siblings can prove a critical test of this hypothesis. However, early markers of ASD identified in prospective sibling studies span a broad range of domains that include both social (Chawarska et al., 2013; Elsabbagh et al., 2012), and non-social abilities (Elison et al., 2013; Elsabbagh et al., 2013b; Flanagan et al., 2012). We discuss the challenges this picture presents for models that place a strong emphasis on early social deficits. Secondly, we identify several methodological improvements that should be considered as the field moves forward, and that are equally relevant to longitudinal studies of any developmental disorder. These include: (i) strategies to deal with publication bias when evaluating evidence for and against particular theories of ASD development; (ii) the need to move away from identifying deficits on particular tasks and toward employing multiple measures of underlying core constructs; (iii) the power of studying the effects of theoretically motivated early intervention programs; (iv) the need to identify not only markers for an ASD diagnosis, but for also predictors of the significant heterogeneity in developmental skills of children in this group and in the wider cohort; (v) the need to consider the specificity of markers to ASD versus other potentially comorbid conditions such as ADHD, and the generalizability of markers to infants from other risk groups; (vi) the need to characterize ‘outcome’ in a consistent way across the field, with particular emphasis on how we characterize children who experience other types of developmental delay.

### 1.1 Approach

We have focused on studies of infants at high familial risk for ASD, who have been followed to ‘outcome’. Since this review focuses on prediction of clinical outcome, effects of risk group (familial high-risk sibs versus low-risk controls) are only discussed where there is outcome data available for that sample. In addition, in order to focus on factors that may contribute to the emergence of the clinical syndrome, we focus on effects occurring prior to the child's second birthday, because relatively reliable diagnosis of ASD is increasingly achievable from age 2 (e.g. Lord et al., 2006).

Of note, studies have used a range of different criteria for ‘outcome’, ranging from the application of gold-standard diagnostic criteria for ASD at 36 months (including administration of a semi-structured observational assessment, parent report of developmental history, and expert clinical judgment) to scores on one observational instrument at age 24 months. We have included all studies with 24-month or 36-month outcome data in this review, because this provides a broader view of the field. However, this may be a key methodological variable that contributes to variance in early markers across studies. Some children with milder symptoms of ASD may not be detected at 24 months, but may receive a clinical diagnosis at 36 months (Cox et al., 1999; Stone et al., 1999). Further, the use of a cut-off on one observational instrument is likely to produce poorer sensitivity, specificity and stability than gold-standard diagnostic procedures (Lord et al., 2006; Risi et al., 2006). In the text, ‘diagnosis of ASD’ refers to studies using at least one instrument and expert clinical judgment of the presence of ASD; ‘observational/parent report of symptoms of ASD’ refers to outcome data that is based on one instrument. More specific details of the criteria used in each study can be found in Table 1 (column headed ‘outcome specification’), and we return to the broader issue of outcome classification in the discussion.

The review is organized in subsections that focus on different symptom areas (social interaction, communication, restrictive and repetitive behaviors, and other symptoms). Within each section, we first summarize key diagnostic symptoms of ASD in that domain; we then outline normative development in that domain in the early years of life; and finally we examine how development progresses within high-risk infants with ASD or other atypical outcomes.

## 2 Emerging evidence

### 2.1 Social interaction

Qualitative impairments in social interaction are a core diagnostic feature of ASD, and include the use of nonverbal behaviors (e.g. mutual gaze, facial expression, posture and gestures) to regulate social interaction; failure to develop peer relationships; lack of spontaneous seeking to share enjoyment or interest (e.g. showing, giving and pointing); and lack of social or emotional reciprocity (e.g. not participating in simple social play). Deficits are usually seen in both initiation and response to social interaction; we will review these areas separately.

#### 2.1.1 Initiation of social interaction

##### 2.1.1.1 Typical development

Infants use communicative and emotional cues to regulate interaction from the first months of life (Trevarthen and Aitken, 2001). Around 8–10 months, typically developing infants begin to use gestures like pointing and showing to initiate episodes of shared attention (Bates and Dick, 2002). The onset of pointing is believed to follow a period of motor development and imitative learning but also to correspond to infants growing to understand that pointing is a means to orient attention and to request things or information (e.g. Begus and Southgate, 2012). From around 9 months, typically developing infants also use gaze to initiate episodes of joint attention, alternating their direction of gaze between a person and an object in order to share engagement (Carpenter et al., 1998). Infants also begin to participate in simple social games like peekaboo or ball rolling at a similar age (Hodapp et al., 1984). Parallel play with other children emerges around the first birthday, with truly co-operative play with peers not common until later in the preschool period (Cohen, 2000).

##### 2.1.1.2 Younger siblings of children with ASD

Contrary to the expectations of many researchers in the field, 6-month-old infants who later develop ASD appear to use communicative and emotional cues to regulate simple interactions relatively successfully (Rozga et al., 2011; Young et al., 2009). These skills can be assessed during a ‘still-face’ procedure, in which the mother briefly stops responding to the infant during a period of naturalistic interaction. This paradigm is sensitive to perturbations of interaction seen across a range of risk groups, including infants with parents with depression (reviewed in Mesman et al., 2009). However, two studies from the same cohort of infants at-risk for ASD have observed no significant differences in gaze or affect in this paradigm in 6-month-old infants who go on to be diagnosed with ASD at 24 or 36 months (Rozga et al., 2011; Young et al., 2009). Young et al. (2009) observed typical patterns of gaze to the mother's face, including decreased attention to mother during the non-responsive phase, in all three infants later diagnosed with ASD. Patterns of gaze and affect at 6 months were also not related to continuous measures of symptom severity at 24 months. Similarly, Rozga et al. (2011) found typical patterns of gaze and affect across outcome groups, with a decrease in gaze to mother and a decrease in smiling during the non-responsive phase. Infants later diagnosed with ASD also showed typical rates of social smiling, social vocalizations and direction of looks, smiles and vocalizations toward mother during a less structured face-to-face interaction. Contrary to predictions of decreased social attention, there was actually a trend for infants later diagnosed with ASD to show greater amounts of gaze to mother's face throughout the task. Taken together, these studies suggest relatively typical engagement in social interaction with mother at 6 months.

However, later emerging skills like the use of gesture appear more disrupted. For example, Rozga and colleagues observed that 12-month-old infants diagnosed with ASD at 24/36 months were less likely to show or point than other high-risk infants and low-risk controls (see also Barbaro and Dissanayake, 2013; Macari et al., 2012), and Landa et al. (2007) report that 14-month-old infants who show signs of ASD and have a 30–36 month diagnosis have a smaller inventory of gestures and are less likely to initiate joint attention than other outcome groups (including infants diagnosed with ASD at 30–36 months but without early signs). Similarly, Talbott et al. (2013) found that infants diagnosed with ASD at 18, 24 or 36 months produced a lower variety of gestures in interaction with mother and an experimenter as infants with other outcomes at 12 months. Yoder and colleagues found that rate of growth in the child's ability to use a combination of gaze, gesture and complex vocalizations between 15 and 24 months predicted 36-month diagnosis of ASD, and level of social impairment (Yoder et al., 2009). Parents also reported reduced use of gesture at 12 and 18 months in infants with a diagnosis of ASD or Autism at 24 months (Mitchell et al., 2006; Zwaigenbaum et al., 2005), and reduced coordination of point and gaze in infants with a community diagnosis of ASD at 36 months (Feldman et al., 2012). Converging evidence for deficits in gesture use in the second year of life comes from early retrospective studies (e.g. Clifford and Dissanayake, 2008; Osterling and Dawson, 1994); suggesting that this may be a replicable early sign of ASD. Since infants use gestures to elicit information from their social partners (Begus and Southgate, 2012), the extent to which reduced use of gesture could contribute to slowed cognitive and social communication development in ASD is a question that can be addressed within the context of longitudinal sibling studies. However, it will also be important to establish whether delays in use of gesture are specific to ASD, or whether they are more generally observed in children with other types of developmental disability.

There is less research on whether and when infants who later develop ASD purposefully use gaze to initiate joint attention, and the findings coming from the prospective literature are mixed. ‘Gaze alternation’ refers to gaze shifts between a person and an object made by an infant for the purpose of drawing the person's attention to the object. Rozga et al. (2011) found that at 12 months, infants with typical and atypical outcomes were equally likely to use gaze alternation to initiate episodes of shared attention, thus finding no evidence of deficits in infants later diagnosed with ASD. Macari et al. (2012) also found no differences in the use of gaze to initiate a joint attention episode in 12-month-old infants diagnosed with ASD at 24 months compared to those with other atypical outcomes, but both groups showed less initiation than typically developing infants. Finally, Landa et al. (2007) found that 14-month-old infants with a diagnosis of ASD at 30–36 months were less likely to use gaze alternation than all other outcome groups. Thus, by 14 months there is clear evidence of atypicality in the use of gaze to regulate social interaction in infants later diagnosed with ASD. However, it is unclear whether gaze alternation is *typical* at 12 months in infants later diagnosed with ASD (Rozga et al., 2011), or whether it is *equally atypical* in children who are later diagnosed with ASD and those with other atypical outcomes (Macari et al., 2012).

This question is critical, because typical gaze alternation at 12 months would suggest that later atypicalities in using gaze to initiate joint attention are a downstream consequence of other risk factors. In contrast, if gaze alternation is atypical in both children with ASD and those with other atypical outcomes, difficulties with gaze alternation could represent an early emerging cumulative risk factor. Methodological differences between studies make this question hard to answer from present evidence. However, it is important to note that the ‘typically developing’ comparison group tested by Macari and colleagues had IQ scores of over 1.5 standard deviations above the mean of the normative sample. Thus, the reduced gaze alternation in children with atypical and ASD outcomes may in part be driven by the unusually advanced cognitive development of the comparison group in this study. Further work in this area is needed to characterize the relation between the use of gaze to initiation social interaction, and later cognitive and socio-communicative outcomes.

#### 2.1.2 Response to social interaction

##### 2.1.2.1 Typical development

The earliest manifestation of responsiveness to social interaction is the intense interest infants show in faces, particularly that of their mother. Typically developing infants show preferences for faces over shapes, and for their mother's face over a stranger's face, from a few hours after birth (e.g. Bushnell, 2001; Johnson et al., 1991a). This early interest in faces may be the gateway to the development of social expertise, because it draws infants’ attention to key social cues from very early in development.

As infants grow older, they become increasingly able to respond to bids for attention. The ability to *follow* another person's attention using gaze or gesture cues changes both qualitatively and quantitatively over the first year of life (Carpenter et al., 1998). Initially a simple orienting response to the movement in gaze cues at birth (Farroni et al., 2004), gaze following becomes apparently more sensitive to the referential nature of the gaze cue. Unlike their younger peers, 10-month-old infants only follow a head turn when the person's eyes are open (Brooks and Meltzoff, 2005). Six-month-old infants can use gaze cues to orient to targets in their visual field (Senju et al., 2008) but 12- to 14-month-old infants can follow gaze to objects that are out of sight (Moll and Tomasello, 2004). Eight-month-old but not four-month-old infants can use gaze direction to bind audio and visual object properties (Wu and Kirkham, 2010). Orienting to a point/gaze target also significantly increases in frequency and precision between 9 and 12 months (Carpenter et al., 1998; Mundy et al., 2007). Thus, in typical development low-level aspects of gaze/point following emerge earlier in development than sensitivity to referential information.

Typically developing infants also begin to increasingly respond to the emotional cues of others over the first year of life. Contagious crying is present from birth, and is maintained across the first year of life (Geangu et al., 2010). Over the first two months, smiling becomes reactive to social cues in the environment (Emde and Harmon, 1972; Wolff, 1987). Social smiling is typically elicited from around 1 to 2 months, in concert with a more integrative gaze pattern to head, eyes and mouth that may facilitate greater attention to facial expression (Anisfeld, 1982). By 10 months, infant smiling is qualitatively different in response to smiling mother versus unsmiling stranger (Davidson and Fox, 1988). Infants use maternal emotional responses to guide their behavior in ambiguous situations by the latter part of the first year (Walden and Baxter, 1989; Walden and Ogan, 1988), particularly for negative responses (Hertenstein and Campos, 2001; Hornik et al., 1987). Infants show prosocial responses to distress (like helping, sharing or provision of comfort) by at least 12 months, and expressions of concern increase over the second year (Zahn-Waxler, 1992). Thus, there is an increasingly sophisticated response to the emotional states of others over the first years of life.

Typically developing infants also respond to social cues through imitation. Infants can imitate simple facial movements from birth (Meltzoff and Moore, 1977), and can imitate actions on objects from at least 6 months (e.g. Barr et al., 1996; Learmonth et al., 2004). Typically developing infants learn one to two novel behaviors a day through observation and imitation in the second year of life (Barr and Hayne, 2003), making imitation a powerful tool for social learning. Imitation is also important in social affiliation, contributing to the intersubjectivity in early mother–child interaction (Trevarthen and Aitken, 2001). Imitation of words and sounds is also an important means by which typically developing children acquire their vocabulary (Masur, 1995; Masur and Eichorst, 2002). Thus, it is likely that imitation is a key mechanism for early social and cognitive development.

##### 2.1.2.2 Younger siblings of children with ASD

Social attention to an unfamiliar adult appears typical at 6 months in infants later diagnosed with ASD. Ozonoff et al. (2010) coded gaze to faces, social smiling, directed vocalizations and social engagement at 6, 12 and 18 months during administration of a standardized table-top task, and found no significant group differences at 6 months that were related to 36-month ASD diagnosis. Further, in a computerized eye-tracking task using a static face amongst nonsocial distractors, Elsabbagh et al. (2013c) found no group differences related to 36-month ASD diagnosis in orienting or visual attention to the face in either 6- or 12- month-old infants. Of note, both studies suggest a trend toward *increased* looking to faces in infants later diagnosed with ASD. However, Chawarska et al. (2013) found that 6-month-old infants diagnosed with ASD at 24–36 months paid less attention to the face of an adult experimenter in a naturalistic video, in addition to paying generally less attention to the screen, than infants with other outcomes. Understanding the contributions of experimental context to these discrepancies may be critical to evaluating the nature of social attention early in the development of ASD.

Clear deficits in social attention are apparent around the end of the first year of life. By 12 months, Ozonoff et al. (2010) found that high- and low-risk infants with a 36-month diagnosis of ASD showed less gaze to faces and fewer directed vocalizations during a cognitive assessment than infants with a typical outcome, and by 18 months they showed a reduced level of social smiling. Social smiling and gaze to faces showed a progressive decline in frequency of occurrence between 6 and 24 months in the infants diagnosed with ASD; directed vocalizations failed to show a typical rate of increase. Of note, the coding scheme used in this study did not differentiate between behaviors elicited by social communication from the examiner (such as social praise after an activity) and behaviors that were initiated spontaneously by the child. Thus, these trajectories likely reflect a combination of changes in social responsiveness and social initiation. In addition, the context in which social behavior was coded may play a role: by 18 months, infants later diagnosed with ASD also showed poorer performance on the cognitive scale used as a context for social assessment. Experiencing greater difficulty with a task may lead to more negative affect and frustration, impacting the child's likelihood of smiling or vocalizing to the experimenter. Examining the dynamic relations between child, examiner and activity will be an important direction for future work in this area.

Despite these caveats, there is a range of other evidence from other contexts that infants who later develop ASD show reductions in social responsiveness by the end of the first year of life. By 9 months, parents report lower interest in faces and reduced shifting to a person in infants with a 3-year diagnosis of ASD (Feldman et al., 2012). Infants diagnosed with ASD at 36 months also show reduced attentiveness to their mother during naturalistic interaction at 12 but not 6 months, in addition to reduced dyadic mutuality (Wan et al., 2012a,b). Further, deficits in responding to own name (a skill that is emerging by 4–6 months in typically developing infants) also appear to emerge by age 9 to 12 months in infants diagnosed with ASD at 24–36 months, but are unclear at earlier time-points (Feldman et al., 2012; Nadig et al., 2007). Further, Macari et al. (2012) found that 12-month-old infants who were diagnosed with ASD at 24 months show less engagement with a researcher than other high- and low-risk infants during participation in a semi-structured observational measure of ASD symptoms (the toddler module of the Autism Diagnostic Observation Schedule; ADOS-T; Luyster et al., 2009). Since the emergence of social orienting problems appears to occur on the same timescale as difficulties with other aspects of social and communicative behavior, existing data provides no evidence that early problems with social orienting are more primary, and have a cascading effect on the emergence of other social symptoms.

Possibly, diminished reward value of social stimuli may over time fail to reinforce early orienting mechanisms that may be predominantly driven by low-level perceptual mechanisms (Morton and Johnson, 1991). However, initial evidence on the development of affective responses does not support this proposal. Ozonoff et al. (2010) found that differences in affective responses to an examiner emerged between 6 and 12 months in infants diagnosed with ASD at 36 months, on the same timescale as differences in social orienting. Hutman and colleagues have also shown that by 12 months, infants with a diagnosis of ASD at 36 months pay less attention and show less affective response to an examiner in distress than infants with other outcomes (Hutman et al., 2010). Infants with a diagnosis of ASD at 36 months are rated by their parents as less cuddly and less likely to smile during caretaking and play at 14 and 24 months relative to low risk infants, but these differences are not apparent at 7 months of age (Clifford et al., 2013). Lower shared positive affect at 14 months in infants showing signs of ASD at both 14 and 24 months was also documented by Landa et al. (2007), and reduced social referencing at 17–20 months was observed by Cornew and colleagues in infants with a 36-month diagnosis of ASD (Cornew et al., 2012). Thus, it appears from early evidence that social reward and affective responsivity may decline on the same timescale as social orienting, though further work examining the integrity of the social reward brain network is critical.

An alternative hypothesis is that difficulty in processing social information could over time result in a decrease in social reward and orientation as infants struggle to deal with incoming information. There is presently mixed evidence from the few studies to date that have examined more complex social information processing. Elsabbagh and colleagues examined modulation of gaze by endogenous and exogenous cues whilst infants saw videos of people playing peek-a-boo (Elsabbagh et al., 2013a). At both 7 and 14 months, infants diagnosed with ASD at 36 months showed normative patterns of gaze modulation, with more looking to the mouth during mouth movement, and more looking to eyes during eye or hand movement. During peekaboo scenes in which eyes, mouth and hands were moving together, increased mouth scanning at 7 months was related to better expressive language at 36 months (see also Young et al., 2009 for a similar finding). However, excessive gaze to mouth when only the mouth was moving was associated with *poorer* expressive language skills and more socio-communicative problems on the ADOS-G (Lord et al., 2000) at 36 months in the high-risk group. The relation of these finding to preference for audiovisual synchronization noted in toddlers with ASD (Klin et al., 2009) is an interesting avenue for further exploration. However, these results broadly suggest no clear differences in the modulation of face scanning by endogenous or exogenous cues in early development.

Event-related potentials associated with basic face processing also appear typical in 6- to 10-month-old infants who are diagnosed with ASD at 36 months (Elsabbagh et al., 2012). However, in the same study processing of dynamic gaze shifts was impaired. Elsabbagh and colleagues measured the P400 response to gaze shifts, an early brain response to visual stimuli that peaks around 400 ms after a stimulus is presented. Whilst high-risk infants later diagnosed with ASD showed no significant P400 differences between gaze shifts toward and away from the infant, other outcome groups showed significantly enhanced P400 responses when gaze shifted away from them. Although the specificity to social information is unclear, it is possible that the neural response to eye gaze shows atypicalities before behavioral responses to social situations emerge. Interestingly, Elsabbagh and colleagues noted that atypicalities in gaze-shift processing were particularly pronounced in infants who met diagnostic criteria for ASD at 36 months and who had scored above threshold on the ADOS-G at 24 months. Infants who look atypical by 24 months may be earlier and more severely affected than infants who do not display the full phenotype until 36 months (see also Landa et al., 2007).

Difficulty in processing gaze cues may reduce interest in eye gaze over time, because infants do not receive the reinforcement of identifying the gaze referent. Indeed, the onset of deficits in *responding* to referential cues in ASD appears to occur early in the second year of life (Landa et al., 2007; Rozga et al., 2011; Sullivan et al., 2007; Yoder et al., 2009). However, whether these measures predict ASD in particular, or social and communication difficulties in general, seems to depend on task demands. Yoder et al. (2009) found that ability to respond to a mixture of gaze, verbal and point joint attention cues at 15 months predicted diagnosis of ASD at 36 months. However, when examining orienting to a target in response to gaze/point prompts at 14 months, Landa et al. (2007) found that infants considered to have signs of ASD at both 14 and 30 months were poorer at orienting to the correct location than high-risk infants with a typical outcome, but did not differ from infants with other atypicalities. Finally, Sullivan et al. (2007) found that consistent failure to respond to gaze and point cues across two tasks at 14 months predicted 36-month-diagnosis of ASD. Of note, deficits were clearer on tasks involving gaze cues only (the more difficult trials). Infant behavior can be strongly affected by state-related variables, and assessing behavior in multiple contexts may provide a clearer signal of impairment. In addition, since greater variability in performance can be a hallmark of acquiring a new skill these findings may reflect developmental delays in response to joint attention in children with ASD.

Patterns of deficit in response to joint attention cues are less clear earlier in development. Rozga et al. (2011) found that 12-month-old infants diagnosed with ASD at 24–36 months showed significantly less success in orienting to a target in response to gaze/point probes delivered during a table-top interaction than infants with other outcomes, but were equally successful at following points to a picture in a book. However, in a computerized gaze-following paradigm Bedford et al. (2012) did not find significant atypicalities in the proportion of correct first look to the target object in either 7- or 13-month-old infants diagnosed with ASD at 36 months. One important factor may be the distance between the cue and the target, which is shorter in pointing to pictures in a book and in the computerized gaze-following task than in the methods used in other studies. Possibly, following a cue to a close, visible target can be accomplished by reflexive orienting mechanisms, which seem typical early in life in ASD, whilst being motivated to search out a more distal location requires an understanding of gaze cues and pointing as place holders for objects in the world. Suggesting that it is the referential nature of the cue that may be difficult for some infants, Bedford et al. (2012) found that at 13 but not 7 months, infants with later socio-communicative problems (those meeting criteria for ASD, those scoring above the ASD threshold on the ADOS-G but not meeting clinical diagnostic criteria for ASD, or with those other delays) spent less time looking at the cued object than infants with typical outcomes. Similarly, Sullivan et al. (2007) noted anecdotally that after following a referential cue, infants diagnosed with ASD at 30–36 months were more likely to look blankly at the target, look at the target more briefly, or fail to look back at the examiner. These behaviors may suggest reduced referential understanding in this group, and indicate that a nuanced consideration of the infant's understanding of joint attention behaviors is important in understanding possible impairments in ASD. However, whether these gaze response behaviors mark later ASD or poor social and communication skills more generally remains unclear.

Since imitation is a key social learning mechanism in early development, early emerging deficits in ASD could contribute to other social and communicative difficulties. Of note, imitation deficits have been observed in toddlers with ASD (Rogers et al., 2003) that predict later language development (Toth et al., 2006). Evidence from studies of high-risk infants suggests that these deficits emerge in the second year of life (Feldman et al., 2012; Macari et al., 2012; Young et al., 2011). By parent report, Feldman et al. (2012) found that imitation of sounds or words at 12 and 18 months was reduced in high-risk infants with a 36-month community diagnosis of ASD, relative to high-risk infants with other outcomes. Further, functional and symbolic imitation in the ADOS-Toddler at 12 months was one of the items that distinguished high-risk and low-risk infants with a 24-month diagnosis of ASD from those with later typical development or developmental delay (Macari et al., 2012). In this probe, infants gain more credit for symbolic and creative use of a placeholder than functional imitation of a previously seen action. Imitation of actions on objects in the semi-structured Autism Observation Scale for Infants (AOSI; Bryson et al., 2007a,b) at 12 months was also reported to predict diagnosis of Autism at 24 months (Zwaigenbaum et al., 2005). However, a recent study found deficits that were not ASD-specific. Young et al. (2011) conducted a longitudinal Rasch analysis of a 10-item imitation battery consisting of three actions on objects, four facial gestures and three hand gestures. Infants diagnosed with ASD and high-risk infants with developmental delays at 36 months showed poorer imitation from 12 to 24 months relative to low-risk controls, but no ASD-specific differences were observed. Thus, it may be that the imitation tasks used by Zwaigenbaum and colleagues are more sensitive to ASD-specific patterns of impairment than the imitation tasks used by Young and colleagues. Alternatively, different analysis methods may reveal different patterns of deficit.

There are many avenues for future work in this area. Systematic study of the developmental progression in imitation from early in the first year is important. Breaking down imitation tasks to ask questions about the origins of impairments is also important – do infants fail to attend to or understand the demonstrated actions? Do they fail to map them onto their own body? Are imitation deficits related to general motor delays? Exploring the longitudinal relation between imitation skill and social affiliation is critical to understanding the potential impact of imitation problems on other aspects of social behavior. Further, investigating social imitative learning is also critical. Examining deferred imitation, imitation generalization and imitation from multiple models is central to understanding how imitation deficits may constrain social learning opportunities in ASD.

### 2.2 Communication

Qualitative impairments in communication that are diagnostic features of ASD include a delay or lack of spoken language with no compensation by other means of communication; impairment in the ability to initiate or sustain a conversation with others; stereotyped or repetitive use of language or idiosyncratic language; and lack of spontaneous pretend or imitative play appropriate to developmental level.

#### 2.2.1 Language

##### 2.2.1.1 Typical development

Language learning begins in the womb, with newborn infants preferring their mother's voice (DeCasper and Fifer, 1980) or a story heard in late gestation (DeCasper and Spence, 1986). Over the first year of life, language input drives increasing specialization to sounds heard in the native language (a process mirrored in social perception; Pascalis et al., 2002). For example, infants whose primary language is Japanese lose the ability to distinguish some English vowel sounds between 8 and 10 months (Kuhl et al., 2006). This process is central to the development of language expertise. Measured using event-related potentials, maintenance of non-native discrimination in the first year predicts vocabulary in the second year in both typically developing and premature infants (Jansson-Verkasalo et al., 2010; Kuhl et al., 2008). By 6–9 months, typically developing infants can recognize the meaning of a few highly familiar words (Bergelson and Swingley, 2012), and by 16 months comprehensive vocabulary ranges from about 92 to 321 words (Fenson et al., 1994). Accelerated vocabulary growth during the second year of life is believed to be a result of various word learning strategies, such as the use of referential cues to establish joint attention, and heuristics like mutual exclusivity or the whole object bias (Carpenter et al., 1998; McMurray, 2007). For expressive language, infants moving through developmental stages of crying and vegetative sounds (from birth), cooing (around 6–16 weeks), vocal play (4–7 months), reduplicative babbling (6–10 months), and nonreduplicated babbling (10–14 months), with first words typical around the end of the first year. By 18 months, infants typically produce around 50 words, although this is highly variable (Fenson et al., 1994), and may begin to produce two word phrases. Of particular relevance to conditions predominately affecting males such as ASD, language development is typically slower in male than female infants (Fenson et al., 1994).

##### 2.2.1.2 Younger siblings of children with ASD

Delays in word learning and vocabulary growth are expected during development in infants who later develop ASD, in part because of the evidence of associations between atypical joint attention and language delays in children with an ASD diagnosis (Charman et al., 2003; Dawson et al., 2004; Sigman et al., 1999; Siller and Sigman, 2008). Several studies have identified delays in receptive language by 12 months of age in infants later diagnosed with ASD. Two studies (Mitchell et al., 2006; Zwaigenbaum et al., 2005) have used the Communicative Development Inventories (CDI) – a parental report questionnaire (Fenson et al., 1993) that compares responses to normative inventories of early words and gestures. Both studies observed that infants diagnosed with Autism or ASD at 24 months understood fewer phrases (e.g. “give me a kiss”; “be careful”) at 12–14 months than other high risk or low risk infants. Several studies have reported similar findings on the receptive language subscale of the Mullen Scales of Early Learning (MSEL; Mullen, 1995), which also tests for understanding of phrases like “give it to me” (with gesture), “give it to mommy” (with no gesture) and “no!” (Landa and Garrett-Mayer, 2006; Zwaigenbaum et al., 2005). Zwaigenbaum and colleagues found lower performance in the infants later diagnosed with ASD relative to other outcome groups at 12 months. Landa and Garrett-Mayer (2006) observed lower scores in infants diagnosed with ASD at 24 months relative to infants with typical outcome at 14 months, although the ASD outcome group did not differ from a language delay group until 24 months. Finally, Ozonoff et al. (2010) found that poorer performance in infants diagnosed with ASD at 36 months relative to those with later typical development emerged between 6 and 12 months, although since this paper did not include an atypical outcome group in the analysis the specificity of these findings to ASD remains unclear. By 18 months, decreased comprehension of single words is added to these earlier emerging difficulties (Mitchell et al., 2006). Differences in single word comprehension are not apparent at 12 months (Mitchell et al., 2006; Zwaigenbaum et al., 2005), perhaps because word comprehension is limited or difficult for parents to accurately report on at that age. However, it may be that rate of word acquisition significantly slows for children with ASD in the second year of life, when advanced word learning strategies become more important. Modeling data from language samples collected frequently across the first years of life would provide insight into this possibility.

In parallel with poor language comprehension, language production is also atypical in infants that develop ASD. A delay in the production of the first words has always been one of the earliest red flags for ASD (Short and Schopler, 1988), and this is born out by high-risk samples. Parents report fewer words produced on the CDI at 18 but not 12 months (Mitchell et al., 2006; Zwaigenbaum et al., 2005). Further, scores on the MSEL Expressive Language subscale differ in 14-month-old infants diagnosed with ASD at 24 months relative to an unaffected outcome group (Landa and Garrett-Mayer, 2006); a trend in this direction is present at 12 months in infants diagnosed with Autism at 24 months (Zwaigenbaum et al., 2005). Suggesting that atypicalities may be present even earlier in development, Paul et al. (2010) observed lower expressive language scores on the MSEL at 6 months in infants who showed high levels of ASD symptoms on the ADOS-T at 24 months. Expressive language skills tested by the MSEL at 6 months include sounds like coos and laughs, vocalizations like ‘ah’ or ‘ah-goo’, imitation of sounds and production of consonants. This finding may suggest that while consistent deficits in word production may not be apparent until later in the second year, there may be earlier emerging atypicalities in underlying foundation skills. However, Paul and colleagues did not observe group differences at 9 and 12 months, so the relation between these early impairments and later word learning difficulties remains unclear. Testing such models is difficult with scores from standardized measures that reflect abilities across a range of domains.

Other studies have identified atypicalities in more subtle aspects of vocal communication. Paul et al. (2010) investigated the phonemic content of speech produced by infants at-risk for ASD. Infant vocal behaviors prospectively associated with high levels of ASD symptoms on the ADOS-T at 24 months were those that had emerged most recently for that age group. Specifically, these infants produced fewer ‘middle’ consonant types at 6 months, fewer ‘late’ consonant types at 9 months, and a lower total number of different consonant types at 12 months than high-risk or low-risk infants with non-ASD outcomes. Thus, these findings may suggest difficulty with each new stage of language production in infants, although the specificity to ASD is unclear. In a study of infant cry samples, Sheinkopf et al. (2012) found that three high-risk 6-month-old infants diagnosed with ASD at 36 months showed cries that were more poorly phonated than those of infants with typical outcomes. Further suggestion of delays in expressive language starting in the first year of life were observed by Iverson and Wozniak (2007) in an intensive study of high and low risk infants tested monthly between 5 and 14 months. Two infants were diagnosed with ASD after age 18 months in the community. One of these infants showed atypicalities in babbling apparent by the second half of the first year, and both showed delayed production of first words (around 18 months). Atypical intonation patterns differentiating infants with a 24-month diagnosis of ASD versus non-ASD were also noted at 12 months by Macari et al. (2012). We do not know whether these atypicalities indicate general compromised motor development or are an early expression of problems with learning language specific phonological or prosodic information, something future studies will have to address. In general, studies should aim to go beyond quantifying language to understanding the source of language difficulties in ASD.

However, not all studies observe early language delays in infants later diagnosed with ASD. Hudry et al. (2013) assessed language understanding with the MSEL, CDI and the Vineland Adaptive Behavior Scales (Sparrow et al., 2005) in high and low risk infants at 7, 14, 24 and 36 months. On the MSEL, infants diagnosed with ASD at 36 months only showed poorer expressive language than high-risk infants with typical (but not atypical) outcomes at the 36-month test point, and did not differ on receptive language scores at any age. No group differences were observed on individual scale scores of the Vineland or CDI at any age; however, reduced receptive over expressive advantage was seen at 24 months in infants later diagnosed with ASD relative to those with typical outcomes. Similarly, Talbott et al. (2013) did not observe reduced expressive and receptive language on the MSEL at 18 months in 9 infants with a diagnosis of ASD at 18–36 months. The absence of early language delays in these studies, coupled with the strong language skills of the high-risk ASD group at outcome, highlights the variability in linguistic abilities within ASD and suggests that measuring early language skills could provide a good predictor for later functioning level. Comparing early language profiles across a larger, more heterogeneous sample of toddlers with an outcome of ASD may provide further evidence as to this possibility. It may also be important to distinguish sub-groups of children with ASD. Landa et al. (2007) used a standardized measure of communicative function (the Communicative and Social Behavior Scales) at 14 months, and found that infants with signs of ASD at both 14 and 30–36 months had fewer consonants in syllables than all other outcome groups. Of note, these differences were not apparent in infants with ASD at 36 months but without early signs at 14 months. Distinguishing groups of children with ASD with different onset patterns may be an important goal for future work, and this point is further expanded in the General Discussion.

### 2.3 Restrictive and repetitive behaviors

Diagnostic features in this domain include preoccupations and restricted interest patterns that are abnormal in intensity or focus; inflexible adherence to routines or rituals; stereotyped and repetitive motor mannerisms; and preoccupation with parts of objects.

#### 2.3.1 Typical development

Repetitive motor behaviors are common in typical development, but generally decrease through the first years of life (Thelen, 1979). Rituals, habits and compulsions increase between age one and two years and decrease again after age four (Evans et al., 1997). For other aspects of the restrictive and repetitive behaviors domain, one difficulty in studying their early emergence in ASD has been the lack of information on areas of typical cognitive functioning that may be relevant. Work on early predictors of restrictive and repetitive behaviors may illuminate relevant domains, allowing further exploration of their typical developmental trajectories.

#### 2.3.2 Younger siblings of children with ASD

Relatively few studies have examined restrictive and repetitive behaviors in infants later diagnosed with ASD. This is however key to addressing whether restrictive and repetitive behaviors emerge as a consequence of reduced engagement with the social world, whether increased object interest interferes with social engagement, or whether the two groups of impairments emerge and develop independently. Loh et al. (2007) analyzed 47 repetitive behaviors and episodes of posturing produced during the administration of a semi-structured assessment (the AOSI) at 12 and 18 months, and found that the only item that distinguished between infants who went on to an ASD diagnosis at 36 months and other groups was frequency of arm waving. Thus, this study suggests that the kinds of behaviors that might be characteristic of older children with ASD may not be present, or may be hard to distinguish from typical patterns, earlier in development.

Providing further evidence for a lack of specificity of repetitive behaviors in the second year, Damiano et al. (2012) coded body-related and object-related repetitive behaviors exhibited during a semi-structured social interaction (the Screening Tool for Autism in Two-Year-Olds; Stone et al., 2000) with 12- to 23-month-old infants at risk for ASD. Infants diagnosed with ASD at 2–4 years did not differ from other high-risk infants on the rate at which repetitive behaviors were produced, but did show differences in the inventory of object relative to body-related behaviors, unlike other high-risk infants. Christensen et al. (2010) found that 18-month-old high-risk infants diagnosed with ASD at 36 months showed fewer functional and more non-functional repetitive play behaviors than low-risk typically developing infants during a free play session that included dolls, blocks and kitchen equipment. These results could reflect disruption of typical play by intrusive repetitive behaviors, and/or engagement in more repetitive play because of limited functional/symbolic play skills. Importantly, controlling for verbal (but not nonverbal) mental age meant that findings became non-significant. Since early functional and symbolic play atypicalities and language impairment in ASD are thought to reflect common underlying symbolic deficits (Lewis, 2003), this may suggest that the ASD-specific differences were driven more by functional play impairments than intrusion of repetitive behaviors. However, future work would be required to confirm this possibility.

Results from another study suggest that examining exploratory behavior may be fruitful. Ozonoff et al. (2008a) analyzed typical (shaking, banging, mouthing, throwing) and atypical (spinning, rolling, rotating and unusual visual exploration) behaviors during an object free-play session in 12-month-old high- and low-risk infants. Children diagnosed with ASD at 24 or 36 months showed at least one atypical behavior (most commonly unusual visual exploration) that was more than 2 standard deviations above the mean of group of children with a typical outcome. The atypical behaviors measured in this study overlap with the repetitive object-related behaviors that did not predict ASD in the study conducted by Damiano et al. (2012), which include spinning, rocking, rolling, collecting, swiping, rubbing, moving, lining, and clutching. Comparing the two studies may suggest that focusing on atypical rather than repetitive exploratory behaviors may be important. In addition, Ozonoff and colleagues also included atypical visual exploration in their list of atypical object behaviors, and this was the most commonly observed behavior in the group who went on to ASD. Examining other aspects of basic visual processing in the early development of ASD may be central to exploring the relevance of this observation.

Thus, although there is some evidence for early atypical exploratory behaviors in ASD, Ozonoff et al. (2008a) acknowledge their current limitations in drawing a conclusive causal developmental story: “If infants with ASD do not fully participate in interactions that are a source of rich sensory experiences, perhaps they seek out other avenues for sensory stimulation” (p. 7). To address this question, we need to examine precursors of these behaviors in the first year. However, it is yet unclear what cognitive mechanisms are under scrutiny. Atypical object manipulation could reflect differences in visual or tactile perception as well as in fine motor skills, all of which could be impaired in ASD. Suggestive of possible early differences in perception, parents report increased sensitivity to low intensity stimulation in 6-month-old infants with an ASD diagnosis at 36 months relative to infants with typical development (Clifford et al., 2013). Further, parents report infants with a 24-month diagnosis of Autism as showing more frequent and intense distress reactions to a variety of stimuli at 12 months than other high-risk or low-risk infants (Zwaigenbaum et al., 2005). Possibly, these sensitivities relate to enhanced discrimination of weak stimuli, which would be consistent with the proposal that individuals with ASD show enhanced perceptual functioning (Mottron et al., 2006). However, more rigorous laboratory testing of discrimination abilities are required to distinguish between enhanced discrimination and increased reactivity (see Keehn et al., 2013); promising work with high risk infants has indicated a group tendency for enhanced sensitivity of the visual M pathway (McCleery et al., 2007), but relation to ASD outcome has not been reported. However, other parents report *faster* adaptation to new stimulation in 6- and 12-month-old infants diagnosed with ASD at 36 months versus those who develop typically, though this effect was reversed by 36 months (del Rosario et al., 2013). Others have noted that some infants with ASD appear to have particularly ‘easy’ temperaments (greater passivity) early in infancy, while others appear to be particularly fussy (e.g. Bryson et al., 2007a). The extent to which variability in these early temperamental features is related to later restrictive and repetitive interests or to symptoms of ASD more generally is also an important question.

### 2.4 Other symptoms

Although not currently included in diagnostic features of ASD, difficulties in other domains such as executive functioning and motor development are common.

#### 2.4.1 Executive functioning

Executive functioning refers to a suite of ‘higher-level’ cognitive functions that underlie flexible goal-directed behaviors, and that share the need to disengage from the immediate environment in order to guide action (Hill, 2004). This domain includes skills like inhibition, rule learning, flexibility and working memory, and is thought to be mediated by the frontal lobe (Alvarez and Emory, 2006). Executive functioning deficits are present in many individuals with ASD, although their specificity, universality and age of onset have been questioned (for review see Hill, 2004; Kenworthy et al., 2008; Rommelse et al., 2011). However, studying executive functions in at-risk populations may address several key questions in the field. First, the mixed evidence for the presence of executive functioning deficits in young children with ASD relative to children with developmental delays (e.g. Griffith et al., 1999; Jones et al., 2013) raises the possibility that any ASD-specific deficits emerge as downstream consequences of other problems. Examining executive function in infants at risk has the potential to address this question. Second, studies with high-risk samples that are at risk for a range of neurodevelopmental outcomes can shed light on the extent to which executive functioning skills operate as a general protective factor rather than a specific risk factor for ASD (Johnson, 2012).

##### 2.4.1.1 Typical development

Executive functioning capacities emerge slowly in the first years of life, driven by the prolonged postnatal anatomical development of the frontal cortex (e.g. Casey et al., 2000). The emergence of executive functioning in typical development has commonly been measured with behavioral tasks adapted from the animal literature, such as the delayed non-matching to sample (DNMS; e.g. Diamond et al., 1999) or A-not-B task (Diamond, 1990). These paradigms show gradual improvement in the ability to extract a rule from a dissociated reward (e.g. Diamond et al., 1994, 1999) or to inhibit a prepotent response over increasing delays (e.g. Diamond, 1985; Fox et al., 1979) over the first and second years of life. Of note, both these paradigms have been used with young children with ASD, although evidence for impairments is mixed (e.g. Dawson et al., 2002; Griffith et al., 1999; Jones et al., 2013).

Tasks requiring a behavioral response like the A-not-B and delayed non-matching to sample tasks are not solved successfully by toddlers until their second year. However, visual attention measures have revealed earlier emerging skills that may be related to executive functioning. For example, working memory can be measured by at least 6 months in an oculomotor delayed response task (Gilmore and Johnson, 1995), though it develops in capacity and duration across the first years (Reznick et al., 2004). Holmboe and colleagues developed a visual attention task to measure inhibitory control, in which infants were required to inhibit saccades to peripheral distractors (Holmboe et al., 2008). Infants were able to successfully perform the task at 9 months, and individual differences in performance were related to performance on the same task at age months. Thus, measures of visual attention may reveal important individual differences in the integrity of early emerging aspects of executive functioning in the first year of life.

Posner and Rothbart (2000) and Rothbart et al. (2003, 2011) have argued that emotional and behavioral regulation skills in infancy may also reflect early-emerging executive skills. Regulation skills can be assessed with observational ‘frustration tasks’ in batteries such as the LabTab (Gagne et al., 2011), or using parent questionnaires such as the Infant Behavior Questionnaire (IBQ; Gartstein and Rothbart, 2003), and generally improve over the first two years of life. One specific ability that may be linked to regulation in very early development is the ability to shift attention between objects, because it is this ability that allows infants to move their attention away from over-arousing situations. For example, greater flexibility of orienting at 4 months is associated with lower parent-reported negative emotionality and greater soothability (Johnson et al., 1991b). Over their first months of life infants go through a series of changes in their ability to plan saccades to peripheral stimuli. Younger infants difficulties with disengaging from central fixations (sometimes referred to as “sticky fixations”; Hood, 1995) gradually give way to faster reaction times. Evidence for frontal or parietal modulation of visual saccades was documented from 12 months of age in typically developing infants (Csibra et al., 1998, 2000), providing further evidence for potential links with executive functioning capacities.

##### 2.4.1.2 Younger siblings of children with ASD

Little data on the emergence of executive functioning skills has been reported in high-risk populations. No studies have yet reported data on the A-not-B and DNMS tasks; since these tasks have been widely characterized in both typically developing infants and young children with ASD and have been linked to specific brain structures in work with rodents and nonhuman primates, this may be one direction for future work.

Measures of visual attention have provided potential evidence of impairment in some aspects of executive functioning. With Holmboe's freeze-frame’ task (Holmboe et al., 2008), Elsabbagh et al. (2011) found that high-risk infants who were less distracted by peripheral stimuli when fixating a ‘boring’ stimulus showed higher levels of ASD symptoms on the ADOS at 36 months. Distraction during the presentation of an interesting stimulus was not predictive. The task used is associated with genes regulating dopaminergic neurotransmission in the frontal cortex (Holmboe et al., 2010), suggesting that this may form an early marker of compromised ‘executive functioning’ in these infants. Since it has been recently proposed that executive functioning may be a protective factor rather than a specific risk factor for ASD (Johnson, 2012), exploring the role of early executive functioning skills in both high- and low-risk infants and in relation to a range of clinical outcomes will be important.

Parent ratings of infant temperament domains thought to be related to emerging regulatory skills have also been studied in high-risk populations. Clifford et al. (2013) observed lower parent ratings of Orienting/Regulation on the IBQ-R at 12–15 months (but not earlier) in infants diagnosed with ASD at 36 months than in low-risk infants or high-risk infants with atypical outcomes, although not in comparison to high-risk infants with typical development. Of note, these group differences were mainly driven by the Cuddliness subfactor, which may reflect social or tactile differences, rather than executive functioning impairments. By 24 months, group differences are clearer but may not be ASD-specific. Clifford et al. (2013) also found that the high-risk group with later ASD was rated as showing less Effortful Control than low-risk infants (though not other high-risk outcome groups). Similarly, Garon et al. (2009) found that infants diagnosed with ASD at 36 months showed poorer effortful emotional regulation than low-risk infants at 24 months, but only marginally significantly differed from high-risk infants with other outcomes. Further, Zwaigenbaum et al. (2005) found that infants diagnosed with Autism at 24 months were rated by their parents as having less inhibitory control than other groups. Feldman et al. (2012) also found that infants who received a community diagnosis of ASD at 36 months were rated by their parents as finding it more difficult to wait to have their needs met at 9, 12 and 18 months, a potential sign of decreased effortful control. These findings may indicate that regulatory control differences only emerge in the second year. However, it is also possible that abilities measured by questionnaires such as the IBQ-R in infancy are not the same as later constructs of regulation or executive control. Gartstein and Rothbart (2003) observed *decreases* in scores on this dimension across the first year, calling into question the face validity of this construct as a measure of executive functioning. Subscales of this domain that decreased with age include duration of orienting to objects (which may decrease over development as processing speed increases), cuddliness (which may decrease as infants become increasingly mobile) and interest in quiet activities (which may decrease as infants become increasingly able to make active choices). Duration of orienting may also be influenced by difficulties in disengaging attention; Zwaigenbaum et al. (2005) found greater duration of orienting to objects in high-risk infants later diagnosed with ASD, consistent with findings of slower disengagement in a lab task (discussed further below). Further, scores on the Orienting/Regulation domain of the IBQ-R are not correlated with scores on effortful control domains of the Childhood Behavior Questionnaire (Putnam et al., 2008), suggesting the two are measuring different capacities. Using lab tasks of emotion regulation may provide more specific information about regulatory capacity in the early development of ASD.

In the more specific domain of attention-shifting, children with ASD appear to encounter similar difficulties with visual disengagement as do very young typically developing infants (Landry and Bryson, 2004). In these tasks a small central animation is used to orient participant's gaze, followed by a peripheral target which is either presented as the central stimulus is removed (*baseline* trials) or while the central stimulus is still on the screen (*overlap* trials). Disengagement is measured as the difference in reaction times between overlap and baseline trials. This led to developmental accounts that have proposed an attentional origin to ASD. According to these researchers, difficulties with disengaging attention could lead to focusing on irrelevant aspects (e.g. the hair and not the eyes, within a face or background objects and not people) and thus missing out on important social information (Landry and Bryson, 2004).

Two studies to date that have investigated disengagement of attention in infants followed up to diagnosis of ASD find no group differences in latencies to disengage at 6 months but emerging differences at 12–14 months (Zwaigenbaum et al., 2005; Elsabbagh et al., 2013b). Those infants diagnosed with Autism or ASD at 24–36 months *increased* their latency to disengage from 6–7 to 12–14 months, whilst disengagement latencies in all other groups decreased or remained the same. A more recent study (Elison et al., 2013) found that slowed latencies to shift attention in the overlap condition could be seen by 7 months in infants with high ADOS scores at 24 months. Of note, this study also observed slowed orienting in a ‘gap’ condition, in which the central stimulus is removed 250 ms prior to the onset of the peripheral target. Whether the impairments at 7 months observed by Elison and colleagues but not in earlier studies reflect greater impairments in attentional orienting in infants with high levels of ASD symptoms at 24 months (in contrast to those who meet diagnostic criteria for ASD at 36 months), or other methodological differences, is a topic for further investigation.

Mechanisms contributing to slowed disengagement in ASD remain unclear. To investigate the neural basis of atypicalities, Elison et al. (2013) examined correlations between saccadic latencies and diffusion tensor imaging measures of brain connectivity. For typically developing infants, slower latencies to orient in the overlap condition were associated with *reduced* radial diffusivity in the splenium of the corpus callosum. The splenium projects to striate and extrastriate visual areas, and to portions of the posterior parietal cortex. Thus, the splenium may play an important role in connections between extrastriate visual areas and frontoparietal attention orienting networks. The authors speculate that reduced diffusivity in the striatum could reflect greater neuronal density, suggesting less progress in pruning and thus an immature state, whilst also noting the many caveats to the interpretation of diffusivity data. Interestingly, slower overlap latencies were associated with *increased* diffusivity in the splenium for infants with high ADOS scores at 24 months, with other high-risk infants showing an intermediate pattern. The authors do not offer an explanation for this reversal of the correlation seen in typically developing infants, but suggest that functional efficiency of the splenium may be an important target for future investigation. Thus, this data suggests that connectivity between visual and attention networks in the brain plays a role in orienting atypicalities in ASD, but further work is needed.

A role for connectivity between visual and attention networks may suggest that reduced top-down modulation of early visual competition contributes to slowed orienting in ASD. Measures of disengagement depend on the relative salience and relevance of the central and peripheral stimuli. For example, typically developing toddlers take longer to disengage from faces, a highly relevant stimulus but toddlers or older children with ASD do not show this differential effect (Chawarska et al., 2010; Kikuchi et al., 2010). In studies reporting slower latencies to disengage from central animations (Elsabbagh et al., 2013b; Zwaigenbaum et al., 2005), effects may be due to excessive engagement with repetitive moving stimuli in ASD; the effect of this interest on visual orienting can be seen in results from the freeze-frame task discussed above (Elsabbagh et al., 2011). Interestingly, the task used by Elison and colleagues employed a mixture of social and non-social central and peripheral stimuli, but too few trials were obtained for each contrast to determine whether orienting was affected by social content; it will be important in future work to disentangle the mechanisms underlying engagement and disengagement of attention by such manipulations.

Keehn et al. (2013) propose a developmental model of ASD in which impairments in attention disengagement compromise a range of other areas of functioning (arousal regulation, perceptual processing, and joint attention), which then in turn contribute to the emergence of ASD symptoms. The authors propose that attention disengagement is a primary impairment in ASD. However, early findings from prospective infant sibling work suggests that disengagement difficulties emerge most strongly at the age that other behavioral symptoms also begin to appear (e.g. Elsabbagh et al., 2013a,b), providing no evidence for primacy of disengagement problems. Further, Bedford et al. (in press) found that impairments in joint attention and disengagement at 14 months made an additive contribution to ASD outcome, rather than one predicting the other. Thus, further work is required to elucidate the precise developmental consequences of difficulties with disengaging attention; examining arousal regulation and perceptual processing may be important steps.

#### 2.4.2 Motor development

Although motor development can often be an area of relative strength for children with ASD, atypicalities have been noted in gross and fine motor coordination and in movement patterns during locomotion and goal-directed motion (for review, Bhat et al., 2011; Fournier et al., 2010); of note, these deficits are not only restricted to children with poor cognitive skills (Jansiewicz et al., 2006). Retrospective home-video studies suggest that motor problems are apparent early in development and include asymmetries in posture, abnormal muscle tone and delay in motor milestones (e.g. Esposito and Venuti, 2009; Ozonoff et al., 2008b; Teitelbaum et al., 1998), though similarities in early pattern between ASD and children with general developmental delays may suggest that early motor impairments or delays are more general signs of compromised neurocognitive development than specific to ASD (Ozonoff et al., 2008a).

##### 2.4.2.1 Typical development

Typically developing infants progress through a series of gross motor milestones that are achieved within relatively narrow time-windows, including rolling (3–4 months), independent sitting (5–7 months), crawling (7–9 months; omitted in some infants), and walking (10–15 months). Similarly, fine motor milestones include development of the pincer grip (9–12 months) and ability to point (8–14 months). Motor development is closely intertwined with social and cognitive development, with attainment of motor milestones often preceding changes in aspects of cognitive function. For example, onset of crawling is associated with developments in memory and spatial awareness (e.g. Campos et al., 2000; Herbert et al., 2007; Clearfield, 2004); onset of walking is associated with more mature bids to share attention (Karasik et al., 2011); and gestural development is clearly associated with ability to communicate (Iverson and Goldin-Meadow, 2005; Iverson, 2010). Postural control is associated with self-exploratory behaviors (Rocha and Tudella, 2008), reaching (Thelen and Spencer, 1998), and hand function (Samsom and de Groot, 2000). Delayed attainment of key motor milestones like rolling, sitting, and independent locomotion can be an important warning sign for a range of developmental disorders.

##### 2.4.2.2 Younger siblings of children with ASD

Several studies have provided suggestive evidence of early motor delays in infants who later develop ASD (Bryson et al., 2007a; Flanagan et al., 2012; Iverson and Wozniak, 2007). In an early case series, Bryson et al. (2007a) observed that four of nine 6-month-old high-risk infants diagnosed with ASD were judged by examiners to have limited motor control. Further, Iverson and Wozniak (2007) noted that both of two infants later diagnosed with ASD were delayed in the onset of walking (15 and 16 months). In a larger study, Flanagan et al. (2012) assessed postural control during a pull-to-sit task in 6-month-old high-risk infants. Infants typically develop the ability to keep their head in line with their body when pulled to sit by around 4 months, and by 6 months head lag is very uncommon in typical development (Bly, 1994). Flanagan and colleagues found that high-risk infants later diagnosed with ASD showed head lag significantly more frequently (present in 9/10 infants) than other high or low-risk infants (7/13 with delays, 6/17 without delays and 7/21 low-risk). This raises the possibility that this measure could be used as a marker for children requiring early intervention, although it is important to note there would be many ‘false positives’. It is important to explore in future work whether this finding reflects abnormalities in the motor system (e.g. poor muscle tone or postural stability), or failure to anticipate the experimenter's actions in pulling the child to sit. Interestingly, Zwaigenbaum et al. (2005) noted that parents reported lower activity level at 6 months in infants later diagnosed with ASD relative to other groups; it is possible that postural stability issues could contribute to this finding. Of note, the presence of head lag is an early predictor of a range of developmental disabilities (e.g. cerebral palsy; Barbosa et al., 2005; Samsom et al., 2002); it is possible that whilst head lag itself is a general marker for compromised neurocognitive development, the underlying reasons differ by condition.

Several groups have examined performance on standardized measures of motor development. The MSEL has scales assessing both fine and gross motor abilities. Landa and Garrett-Mayer (2006) observed significant delays at 14 months in both fine and gross motor skills in infants diagnosed with ASD at 24 months relative to unaffected infants, though they did not differ from infants with language delays until 24 months. Ozonoff et al. (2010) examined trajectories of scores on the Fine Motor scale of the MSEL and observed group differences that became significant between 12 and 18 months. However, Leonard et al. (2013) did not observe any ASD-specific patterns of motor delay on the MSEL at 7, 14, 24 or 36 months; these findings were mirrored on the parent report Vineland. Thus, evidence for early motor delay from standardized measures is mixed; possibly, delays emerge with age, or may be specific to a subgroup of children with ASD (Landa et al., 2012). Of note, Leonard and colleagues did observe generally poorer performance in the high- than low-risk infants across all time-points. However, other studies have combined high and low risk infants in forming outcome groups, such that most of the ASD outcome group comes from the high-risk sample and most of the unaffected group comes from the low-risk sample (e.g. Landa and Garrett-Mayer, 2006; Ozonoff et al., 2010). Risk group differences may thus confound interpretation of outcome group differences in these studies.

## 3 Conclusions and recommendations for future work

The present review highlights several theoretical and methodological implications for work with infants with older siblings with ASD, in addition to identifying many potentially fruitful avenues for further investigation.

### 3.1 Theoretical implications

Theories of developmental causal paths to ASD seek to identify core deficits that emerge prior to other clinical symptoms. The high-risk literature provides a critical test of such theories.

#### 3.1.1 Social orienting and social motivation

Several influential accounts have proposed that reduced *social orienting* or *social motivation* during early development could underlie many of the deficits seen in ASD (Chevallier et al., 2012; Dawson et al., 1998; Mundy and Neal, 2000). Since in typical development social orienting mechanisms are functional in the first months of life, social orienting accounts of ASD predict very early expressions of risk. This is currently not supported by the literature reviewed above, although it is important to note that there are currently very few studies in the first postnatal months. Just like typically developing infants, infants who later develop ASD orient to faces (Elsabbagh et al., 2013c; Young et al., 2009), and to social movement (e.g. eyes, mouth or hands movement, Elsabbagh et al., 2013a). Gaze following to close targets is also typical during the first year of life (Bedford et al., 2012). Early enjoyment of social interaction seems to decline on the same timescale as social orienting for infants who later develop ASD, providing little evidence that social reward is impaired early in development (Clifford et al., 2013; Ozonoff et al., 2010). Current evidence thus suggests that the mechanisms that underlie social orienting in very early development are intact in the early development of ASD, ensuring exposure to relevant social information during the first year of life.

Other theories that place emphasis on early atypicalities in social functioning have been less extensively tested. For example, Pelphrey and Carter (2008) propose that atypicalities in the modulation of neural activity in the superior temporal sulcus (STS) in response to social stimuli could be a key neural deficit in ASD. Along with a network of other ‘social brain’ regions, the STS is particularly sensitive to cues like biological motion, gaze direction and facial expression. Although young infants with later ASD appear to show typical behavioral responses to these cues, recent preliminary evidence indicates that high-risk infants show attenuated STS responses to social stimuli at 5 months of age (Lloyd-Fox et al., 2013), and infants with a 3-year diagnosis of ASD show atypical neural responses to gaze shifts at 6–9 months (Elsabbagh et al., 2012). Possibly, these atypical early neural responses are related to later atypicalities in behavior, such as reduced preferences for biological motion in toddlerhood (Klin et al., 2009). Longitudinal studies examining STS functioning in infants followed to diagnosis of ASD are currently ongoing, and will provide important information about the potential role of STS functioning in the emergence of ASD symptoms.

If atypicalities in STS functioning are a primary deficit in ASD, why might young infants with later ASD show apparently typical social behavior in the first months of life, given the STS is activated by social stimuli from shortly after birth (Farroni et al., 2013)? Early social orienting is thought to be primarily subserved by a rapid and automatic subcortical neural pathway (Johnson, 2005), providing critical input into the key cortical areas that later play an increasing role in guiding social behavior. If subcortical social orienting mechanisms initially operate typically but are gradually superseded by atypical functioning in cortical areas such as the STS, infants with later ASD may show typical early social behavior followed by a gradual derailment in skills. Indeed, a recent study provides evidence consistent with this proposal (Jones and Klin, 2013). Jones and Klin (2013) examined gaze responses to videos of naturalistic caregiver interactions in 11 high- and low-risk infants with a 36-month diagnosis of ASD tested longitudinally between 2- and 24-months, and a comparison group of 25 low-risk typically developing infants. Analyses showed that whilst typically developing infants showed an increasing tendency to fixate on the eyes over developmental time, eye fixation time declined in infants with later autism. Further analyses indicated that trajectories were significantly different in the 2- to 6-month age range, possibly documenting the gradual erosion of initially typical social orienting skills in the first 6 months of life. Of note, consistent with most previous work cross-sectional comparisons at 6 months did not reveal significant group differences (though see Chawarska et al., 2013). Results of this study could thus be consistent with a model in which atypicalities emerge as cortical systems increase their influence over behavior, although combining measures of behavior with measures of brain function will be an important step to validate this interpretation.

Interestingly, Jones and Klin (2013) found that at 2 months infants with later ASD spent *longer* looking to the eyes than infants with later typical development. Parallels can be found in work on language acquisition in autism, where it has been proposed that atypical attention capture by speech may underlie difficulties in language acquisition (Kuhl et al., 2005). Here, high-risk infants show *increased* response to name at 4 months (Yirmiya et al., 2006), with reductions in response in infants who later develop ASD only apparent by 12 months (Nadig et al., 2007). Asking whether such *increases* in social attention can really be considered ‘typical’, or whether they may provide important clues to the underlying neural mechanisms of emerging atypicality, is an important question for empirical and theoretical work in this area.

#### 3.1.2 Domain-general accounts

The broad range of early markers identified in the high-risk literature so far raise substantial challenges to any account of a primary underlying cognitive deficit. These include declining gaze to eyes between 2- and 6-months (Jones and Klin, 2013); atypical neural response to gaze at 6–10 months (Elsabbagh et al., 2012); presence of head lag at 6 months (Flanagan et al., 2012); reduced interest in faces, attention shifts to person, mood, response to name and waiting by parent report at 9 months (Feldman et al., 2012); higher level of perceptual sensitivity at 7 months (Clifford et al., 2013); production of fewer middle consonant types at 6 months and fewer late consonant types at 9 months (Paul et al., 2010); less looking at a person's face and a social movie at 6 months (Chawarska et al., 2013); slower attention disengagement at 7 (Elison et al., 2013) and 12–14 months (Elsabbagh et al., 2013b), and lower activity level at 6 months (Zwaigenbaum et al., 2005). These observations do not appear to cluster in a particular domain. Possibly, examining children earlier in development (before 6 months) may reveal a more restricted profile of deficit. Alternatively, it may be that focusing on understanding neurocognitive endophenotypes will reveal atypicalities that underlie several behavioral deficits.

One possible domain-general deficit that could underlie many early markers is atypical top-down modulation of perceptual input, resulting from atypical long distance communication between frontal or parietal areas and sensory processing areas. The concept that ASD is characterized by atypical top-down modulation of bottom-up information processing is shared with several theoretical accounts derived from work with older children and adults (e.g. Happé and Frith, 2006; Pellicano and Burr, 2012). For example, Pellicano and Burr (2012) have recently proposed that many of the perceptual differences associated with ASD can be accounted for within a Bayesian framework by attenuated application of ‘priors’, which would result in a reduced tendency to modulate input by prior experience and consequently a more ‘accurate’ view of the world. Cognitive tests of this possibility with high-risk infants using paradigms that require infants to use prior knowledge to modulate behavior or perceptual processing will be an important future goal (e.g. Brooks and Meltzoff, 2002; Gliga et al., 2010); using computational models to test candidate mechanisms underlying the emergence of ASD symptoms in high-risk infants is also an important step toward developing more rigorous theoretical perspectives in this area (e.g. Thomas et al., 2011).

On the neurobiological side of this hypothesis, the possibility of reduced or atypical structural and functional connectivity in ASD has received much attention in the brain imaging literature (e.g. Geschwind and Levitt, 2007; Just et al., 2007; Müller et al., 2011; Murias et al., 2007). In influential work, Just et al. (2007) have proposed that cortical underconnectivity is a general characteristic of the neurobiology of ASD. Underconnectivity may disrupt communication between brain areas, which would particularly disrupt domains of functioning like social communication that require the coordinated activity of networks of brain regions. In a test of the structural aspects of this hypothesis, Wolff and colleagues used fractional anisotropy to investigate white matter development at 6, 12 and 24 months in high-risk infants who scored over the ADOS cut-off for ASD at 24 months (‘ADOS Positive’) versus those who did not (‘ADOS Negative’). In ADOS Positive infants, fractional anisotropy measures showed initial *over*-connectivity at 6 months across commissural pathways (i.e. the corpus callosum) and projection pathways (e.g. the left fornix and the internal capsule). Differences disappeared at 12 months and were reversed at 24 months, such that the ADOS Positive group showed lower connectivity in some pathways (e.g. the anterior thalamic radiation). The fact that this study did not include a low-risk control group means that it is impossible to determine whether these differences reflect risk factors for ASD, or protective factors against ASD symptom development. Nonetheless, whilst the results were consistent with previous work in demonstrating that children with ASD show under-connectivity by the time of diagnoses, in earlier development there is rather a pattern of over-connectivity.

Initial over-connectivity is consistent with Courchesne's model of early brain overgrowth in ASD. Studies of head circumference that include multiplex families have suggested that there may be an accelerated rate of increase in head circumference between 6 and 9 months in infants who later develop ASD (e.g. Constantino et al., 2009; Elder et al., 2007; Hazlett et al., 2005; Webb et al., 2007). Based on such work and other neuroimaging findings in older children, Courchesne et al. (2007) have proposed that there may be early brain overgrowth in ASD that results from excess neuron numbers or insufficient synaptic pruning. This in turn is hypothesized to lead to an excess of short-range connections and the disrupted development of the large-scale, long-distance interactions between brain regions that are critical for social and communication functions. Such accounts have been recently challenged by studies that cast doubt on head circumference findings, suggesting that use of inappropriate norms (Raznahan et al., 2013) and failure to control for body size (Chawarska et al., 2011) represent significant confounds. Rigorous large-scale studies of head circumference growth in high-risk infants versus closely matched controls will provide an important source of evidence in this debate. Nonetheless, consistent with Courchesne's model a recent MRI study found increased cerebral volume at age 12–15 months (but not 6–9 months) in infants later diagnosed with ASD relative to high or low risk infants with later typical development and controlling for body weight (Shen et al., 2013). This is consistent with reports of larger cerebral volume at 24 months in children with an ASD diagnosis (Schumann et al., 2010). Computational models have been developed that link early overgrowth to developmental regression (Thomas et al., 2011); these may provide one way to develop and test theoretical accounts that link imaging findings with the emergence of neurocognitive markers in high-risk infants with later ASD.

Interestingly, Shen and colleagues also observed increased extra-axial fluid at both time-points in infants later diagnosed with ASD; the degree of excess fluid was correlated with later social and communication symptom severity. The authors suggest that this finding could reflect immaturity of arachnoid granulations or poor lymphatic drainage, and could reduce the brain's ability to eliminate harmful metabolites and toxins that are normally excreted by this route. This finding could also contribute to reports of elevated head circumference in ASD, which is the primary clinical indicator of excess cerebrospinal fluid. Thus, it appears that atypicalities are present in a range of interrelated indices of brain development during the first year of life. Examining the relation between these measures in individual infants is an important step toward understanding direction of causality.

#### 3.1.3 Multiple paths to ASD

It is also important to consider that there may be many paths to ASD. Converging evidence from work with older children and adults with ASD suggests that the condition might be better thought of as the ‘autisms’ (Geschwind and Levitt, 2007), and that support for a single underlying cognitive deficit is limited (Happé et al., 2006). Determining whether multiple risk markers are found in the same infants, or whether each deficit is represented in a different subgroup of infants, will illuminate whether risk factors aggregate to produce the clinical outcome, or whether children with a different type of ASD have a different developmental path. Supporting the presence of different developmental paths to ASD, Landa et al. (2012) identify four developmental trajectories in their low and high-risk sample, with children with ASD predominately spread amongst three of them. Similarly, Macari et al. (2012) identify four subgroups of children with ASD with different patterns of early signs at 12 months. Recently, Bedford et al. (in press) also showed that gaze following and attentional disengagement at 13 months made independent and additive contributions to 36 month ASD outcome, consistent with the possibility that gaze following and attentional disengagement problems are present in different subgroups of children with later ASD. Examining the relation between early trajectory, genotype and later phenotype in a larger study would provide important evidence as to whether such subgroups are meaningful at multiple levels of analysis.

Characterizing longitudinal associations can also be critical to examining assumptions that behaviors seen in high but not low risk infants always represent ‘risk’ factors for ASD. Young et al. (2009) found that a face scanning pattern associated with high-risk infants (more looking to the mouth) was actually related to better language development, and increased rates of growth in adaptive socialization skills, across all groups. Of note, increased looking to the mouth was not seen in the infants who later developed ASD within the high-risk group. Possibly, increased looking to the mouth acts as a protective factor when infants find language more difficult to process. Partially replicating these findings, Elsabbagh et al. (2013a) also found that increased looking to mouth versus eyes at 7 months during a “peekaboo” video (in which eyes, mouth and hands were moving) predicted better expressive language at 36 months across a high and low risk group. However, there was no association with later ADOS scores. Interestingly, increased looking to the mouth when only the mouth was moving predicted lower expressive language scores and more social and communication symptoms on the ADOS. This may reflect the additional influence of exogenous parameters like audio-visual synchrony that may be more attractive in ASD (Klin et al., 2009). Examining developmental relationships is key to distinguishing risk markers, protective factors and incidental findings in the early development of ASD.

#### 3.1.4 The role of intervention studies

Examining the influence of early intervention programs is one way to study the causal nature of developmental relationships, in addition to potentially ameliorating or preventing emerging symptoms of ASD (Dawson, 2008). One example from work with toddlers with ASD is the link between joint attention and language development. Several correlational studies had observed that early joint attention skills predict later language development (e.g. Charman et al., 2003; Dawson et al., 2004; Sigman et al., 1999; Siller and Sigman, 2008), and it is intuitive that children who are better at jointly attending with another person are given more opportunities to learn language. Consistent with this prediction, interventions aimed at improving joint attention skills in young children with ASD (e.g. Kasari et al., 2006; Jones et al., 2006) showed a positive effect on both joint attention and language development (Kasari et al., 2008, 2012; Jones et al., 2006).

Joint attention may also play a broader role in the emergence of other complex social behaviors. For example, Mundy et al. (2009, 2010) propose a parallel-distributed processing model in which executive joint attention is fundamental to the development of domains like symbolic thought, social cognition and social competence. Mundy's model adopts a constructivist view of development, placing heavy emphasis on the child's active role in shaping their own experience, and relating cognitive development to connectivity between brain areas. The emergence of joint attention is proposed to be reliant on anterior and posterior attention networks and their connections, theoretically linking joint attention impairments to problems with disengagement (Elsabbagh et al., 2013b) and reduced connectivity (Wolff et al., 2012). Indeed, recent evidence has linked joint attention capacities at 9 months to frontolimbic connectivity at 6 months in typical development (Elison et al., 2013). Examining such relations and their modification by targeted intervention in high-risk infants would indicate whether improving joint attention skills in early infancy might be an important first step toward preventing the emergence of some symptoms of ASD. However, it is important to note that work with high risk infants reviewed in previous sections does pose some challenges to the model, since the apparent emergence of deficits in joint attention between 12 and 14 months occurs on the same timescale as the emergence of language delays and other social difficulties; and joint attention deficits do not appear to relate to deficits in other putative measures of the functioning of attention networks at this age (Bedford et al., in press).

Developing successful intervention programs for infant siblings to test similar questions will likely involve incorporating characteristics of interventions designed for infants with other risk factors (Wallace and Rogers, 2010) and downwards extending intervention programs for toddlers with ASD (Dawson et al., 2010; Rogers et al., 2012). Wan et al. (2012a) showed that aspects of parent–child interaction at 12 months predict diagnosis of ASD at 36 months. The authors propose that this reflects the effect of the child's emerging symptoms on the dynamics of the parent's behavior. This disruption to a natural interaction style may further reduce the child's social learning opportunities. To address this, Green and colleagues developed a video-based intervention that aims to increase parent synchrony (Green et al., 2013). The efficacy of this intervention is currently being tested in a large randomized control trial, which will allow researchers to test whether improvements in interactive synchrony alter the developmental trajectory of at-risk infants. Tests of the efficacy of a developmental adaptation of Pivotal Response Treatment^1^ (Steiner et al., 2013) and Promoting First Relationships^2^ (Webb et al., in press) are also in progress. This work has the potential to move us from markers of ASD diagnosis to identifying causal paths to symptom development, in addition to providing critical information about potential treatments for children with increased risk for developing ASD.

#### 3.1.5 Specificity and generalizability

Examining the specificity and generalizability of early developmental paths to later *ASD* outcome is also critical. Approximately 20% of children with older siblings with ASD will go on to other developmental difficulties such as language delay or subclinical social and communication problems (Messinger et al., 2013). Further, there is significant co-morbidity between ASD and disorders like Attention Deficit/Hyperactivity Disorder (ADHD), and there may be common risk pathways to the two disorders (Rommelse et al., 2011). This raises the possibility that early markers of ‘ASD’ may actually represent early markers for comorbid ADHD, particularly when considering findings on attention (e.g. Elsabbagh et al., 2013b; Elison et al., 2013). Identifying which early markers relate specifically to ASD, and which may predict the ‘broader phenotype’ or represent shared or independent risk factors for comorbid conditions, is important both for clinical identification of at-risk children and for understanding causal paths to symptom development. To date, the field has focused on contrasting groups of children who meet criteria for ASD, ‘other problems’, or typical development. Some studies additionally correlate infant data with scores on instruments like the ADOS. However, the ADOS algorithm scores were not designed to provide a quantitative metric of social and communication symptoms, limiting the validity of this approach. Taking a more rigorous dimensional approach may be necessary to move forward. Examining relations between infant data and childhood phenotype across the whole high risk group using dimensional instruments such as the Social Responsiveness Scale Preschool (Constantino et al., 2003), the Conner's Rating Scale (Conners et al., 1998) or the Restrictive and Repetitive Behavior Scale-Revised (Lam and Aman, 2007) is thus an important avenue for further work.

We must also question whether identified markers are specific to families with multiple children with ASD (‘multiplex’ families). Differences in familial symptom expression (Virkud et al., 2009), infant sibling risk (Schwichtenberg et al., 2010) and genetic copy number variation rates (e.g. Sebat et al., 2007) between multiplex families and those with only one child with ASD (‘simplex’) may indicate that these represent distinct developmental paths to ASD. The apparently low rate of regression in prospective studies of high-risk infants is worthy of note here (Rogers, 2009). Very few cases of frank regression have been reported in high-risk samples, despite estimates of regression in approximately 20 to 30% of children with ASD in other samples (e.g. Hansen et al., 2008; Pickles et al., 2009). More detailed trajectory analysis may reveal gradually declining frequencies of social behaviors in high-risk infants whose parents do and do not report frank regression (Ozonoff et al., 2010). Gradual patterns of skill loss may be more or less noticeable to parents depending on children's starting abilities; indeed, presence of frank regression has been associated with stronger early communication skills (Luyster et al., 2005; Pickles et al., 2009). However, the extent to which work on high-risk infants gives a representative picture of skill loss in ASD symptom development requires further investigation. Prospective studies that make direct comparisons between infants at familial risk for ASD and other risk groups such premature infants (e.g. Cohen et al., 2012), infants with older siblings with ADHD (e.g. Auerbach et al., 2004) or the general population (e.g. Allely et al., 2012; Rai et al., 2012) will be central to characterizing the generalizability of early markers beyond infant siblings.

### 3.2 Methodological implications

Several methodological considerations governing infant sibling research have been outlined by Zwaigenbaum et al. (2007). These include sample size, age of ‘outcome’, selection of appropriate comparison groups, and ethical issues about working with this sensitive population. The current review illustrates the difficulties posed by heterogeneity in factors such as outcome age and comparison group selection within the field. However, several further methodological issues can be identified.

#### 3.2.1 Publication bias

Well-characterized publication biases often favor the publication of positive findings, which in the high-risk literature may mean that studies finding predictive evidence for ASD are more likely to be published than those measures that do not appear to relate to outcome. However, it is clear that publishing results of experiments that did not show predictive effects for ASD is critical. Papers demonstrating typical performance in social orienting tasks (e.g. Elsabbagh et al., 2013c) provide critical evidence against the presence of early social orienting deficits. Since most studies employ multiple measures at multiple time-points, publishing reports on all tasks from a study is critical to evaluating whether particular results have emerged because of the potential for multiple comparisons. One strategy may be for groups to publish ‘mini-reviews’/monographs of findings from all the tasks included in a particular cohort once all data has been individually published. Data repositories such as the National Database for Autism Research (http://ndar.nih.gov/) and research networks such as the Baby Siblings Research Consortium (http://www.autismspeaks.org/science/initiatives/high-risk-baby-sibs) and the EU-AIMS consortium (http://www.eu-aims.eu/; Murphy and Spooren, 2012) can also aid in this endeavor.

#### 3.2.2 Multiple measures of core constructs

As fruitful areas of investigation are beginning to be identified, there is a need to increase the use of multiple measures and methods to assess core constructs. Using multiple measures of a single construct and modeling results using latent variables has proved valuable in work with toddlers with ASD (e.g. Dawson et al., 2004; Munson et al., 2008). This strategy can reduce measurement error and provide more power to detect effects. Further, including neuroimaging or psychophysiological methodologies such as electroencephalography, near infrared spectroscopy, magnetic resonance imaging or electroencephalography to ask similar questions can provide complementary insights into the physiological responses and neural mechanisms that underlie patterns of behavior. This may be particularly important, since atypicalities in neural networks may precede the emergence of overt behavioral signs of ASD (e.g. Elsabbagh et al., 2012; Wolff et al., 2012). However, it is important to recognize that experimental and neurophysiological assessments should also be subject to the same rigorous evaluation of reliability and validity as standardized behavioral assessment, a process that is currently uncommon in this literature (see Bishop, 2013 for a discussion of this issue in the clinical trials domain).

Testing fewer domains will also allow for more intensive sampling, an approach that is critical to properly understanding the shape and nature of developmental trajectories (Adolph and Robinson, 2008, 2011). For example, Adolph and Robinson (2008) asked 32 parents to report on their typically developing infant's motor behaviors daily from birth to 18 months. This revealed significant within- and between-infant variability in skill acquisition. For example, whilst one infant made 21 transitions between meeting criteria for standing and not meeting those criteria, another infant made only one transition. Decreasing the frequency of sampling in one-day steps resulted in a precipitous drop in sensitivity to this variability, such that these infants were indistinguishable when sampling occurred at a 2-week interval. Recent evidence concerning increased variability in neural response in older children and adults with ASD (e.g. Milne, 2011; Dinstein et al., 2012) and suggestive evidence of delayed motor milestones in high-risk infants who later develop ASD (Bhat et al., 2012; Iverson and Wozniak, 2007) indicate that characterizing individual variability in motor skill development is important.

#### 3.2.3 Parsing heterogeneity

Where methods have been repeated across samples, results can vary. For example, Zwaigenbaum et al. (2005) and Clifford et al. (2013) have used the Infant Behavior Questionnaire to explore early temperament differences. While Zwaigenbaum and colleagues find that decreased activity level at 6 months, increased intensity of distress and duration of orienting at 12 months predict ASD at 24 months, Clifford and colleagues find that increased perceptual sensitivity at 7 months, and increased negative affect and decreased cuddliness at 14 months predict 36-month ASD diagnosis. Similarly, whilst some groups have reported significant delays on the MSEL by 12 months (e.g. Zwaigenbaum et al., 2005) others have not (e.g. Leonard et al., 2013; Hudry et al., 2013). Methodological and sample characteristics likely contribute to these differences, and these findings illustrate the importance of following early reports with replication in larger samples.

Examining subgroups based on early trajectories may be one way to understand heterogeneity. Landa et al. (2012) used latent class analysis to examine early developmental trajectories in a group of 204 infants with older siblings with ASD between 6 and 36 months. Infants who later developed ASD (*n* = 52) were spread over four classes, representing (i) infants with accelerated early development (2% of ASD outcome group); (ii) infants with normative early development and above-average nonverbal cognitive outcome (25% of ASD outcome), (iii) infants with receptive language, gross and fine motor delay (31% of ASD outcome), and iv) widespread delayed skill acquisition (42% of ASD outcome). This study illustrates the heterogeneity in early pathways to ASD, and underlines the importance of considering individual differences within groups of infants who later develop ASD. Studies reviewed above with seemingly contradictory findings may have sampled different proportions of these subgroups. Examining whether children in different onset trajectory subgroups have a different pattern of genetic or environmental risk factors for ASD is also an important future step toward determining whether heterogeneity can be linked across multiple levels of analysis.

Understanding trajectories of symptom development is important not only theoretically but also clinically, since being able to predict later severity may help parents to decide how to divide treatment resources between their older child and their infant. However, it will be important to continue to map trajectories beyond age 3. Although diagnosis is relatively stable by this age, children have very different trajectories in the development of cognition, social and communication skills across childhood (e.g. Munson et al., 2008; Fountain et al., 2012; Siller and Sigman, 2008). Some children with ‘optimal outcomes’ appear to move off the spectrum (Fein et al., 2013; Kelley et al., 2010), whilst other children will continue to have poor adaptive skills that compromise their ability to live independently (Charman et al., 2011a,b). Characterizing causes and predictors of this significant heterogeneity is important, because treatment resources should be concentrated for those individuals in greatest need. Many individuals with ASD with less severe symptoms prefer support and environmental adaptation to intervention or treatment (Harmon, 2004), and this should be taken into account when considering the ethics of early intervention.

#### 3.2.4 Outcome diagnosis

Few studies have identified early predictors of restrictive and repetitive behaviors in infancy. One possible barrier is the lack of clarity on the typical development of behaviors and cognitive processes relevant to this domain. Further work on topics like sensory sensitivity and development of circumscribed or special ‘interests’ in the typically developing literature would be of use here. A further issue may be the fact that a DSM-IV diagnosis of PDD-NOS (which would be included in the ‘ASD’ outcome group of most current studies) does not require children to exhibit restrictive and repetitive behaviors. This may make it more difficult to identify early predictors of these behaviors than in the social and communicative domains, for which all children with an ASD outcome will display deficits. DSM-5 requires children given the label ASD to display patterns of restrictive and repetitive behaviors, removing children with only social and communication delays from this group. Possibly, grouping children at outcome using the new classification system may result in samples in which restrictive and repetitive behaviors are apparent at an earlier age.

A second issue related to outcome diagnosis is that agreement within the field about appropriate grouping strategies is vital. Whilst most groups use similar criteria to identify infants who later develop ASD, the characterization of infants who develop typically versus those with other atypical outcomes varies widely (see Table 1). Developing a consensus on how to classify these infants, or turning to more widespread use of continuous metrics of performance in particular domains, is important to begin to synthesize information across the field. Another related issue is analysis strategy. The observation that some effects seen in high-risk infants do not translate to outcome-group effects (e.g. Leonard et al., 2013; Hudry et al., 2013; Young et al., 2009) indicates the critical importance of differentiating between risk group and outcome group effects. It is important to recognize that risk group effects cannot be assumed to reflect genetic vulnerability for ASD; the significantly higher rates of maternal psychopathology in families with a child with ASD (e.g. Carter et al., 2009; Taylor and Warren, 2011; Weitlauf et al., 2012) and the known effects of maternal psychopathology on infants’ development (e.g. Field, 2010; Goodman et al., 2011) make this one potentially significant source of risk effects that do not translate to outcome, and should be explored within high-risk samples. To avoid confounding risk and outcome group effects, researchers should thus compare infants who later develop ASD (who almost always come from the high-risk group) with other high-risk infants with other outcomes. Including low-risk controls is however still critical to evaluating whether patterns seen in high-risk infants with typical outcomes might represent protective factors. Studies that contrast high-risk infants who later develop ASD with low risk infants with typical development, or who do not include low-risk control groups, thus contain significant confounds.

#### 3.2.5 Building capacity

Whilst we have identified many methodological and theoretical considerations for future work, it is important to recognize their significant resource implications. Testing a sufficient sample of infants from early in development to 36 months (the age at which many scientists regard diagnoses of ASD to be relatively stable) generally requires project durations that exceed the typical 5 year maximum of standard grants. Progress in this field is thus necessarily slower than is typical of cross-sectional research approaches. Intensive sampling and using multiple methodologies requires a large time-commitment on the part of families, which may increase the bias toward the inclusion of families who have the resources to participate in such research. Continuing to involve parents and other stakeholders in dialogs about acceptable levels of burden is critical to effective work in this area. Developing low-cost options for families who may find participation more difficult is another key goal for the field; this may also allow investigators with more limited resources to collaborate on joint work to increase sample sizes. We are currently developing such a low-cost protocol that can be completed by parents in their home as part of a European collaborative initiative to build capacity for early ASD research in Europe (http://www.cost-essea.com/).

Attracting early-career researchers to the field is also a challenge. Significant questions cannot be answered until a cohort of children reach ‘outcome’ age, but this may exceed the timescale of a typical PhD or postdoctoral position. Nonetheless, inclusion of early career scientists in this field is key to building capacity for such work in the future. Data sharing provides one platform for scientists to capitalize on unpublished datasets from labs who have been working in this area for longer. The British Autism Study of Infant Siblings (BASIS) network (http://www.basisnetwork.org/) and the Baby Sibs Research Consortium (BSRC) provide excellent examples of the power and practice of data sharing in this field. However, whilst effective mechanisms exist for sharing clinical data, such efforts are further behind for experimental methods such as electroencephalography, near infrared spectroscopy, or eye-tracking. Data collection systems, paradigms and procedures must all be harmonized before such efforts can reach their full potential, an endeavor that is currently ongoing within the EU-AIMS project. Finally, data sharing efforts can be complicated by the scientific career structure, which still places heavy emphasis on publication record. If data is too freely shared, this can create a disincentive for investigators to place significant time resources into collecting data because publications can be more easily gained by secondary analyses. These issues are complex, but the development of new paradigms for career evaluation that increase the likelihood of data sharing could be broadly beneficial to progress in this and other fields.

### 3.3 Summary

The early wave of studies of infants with older siblings with ASD has revealed a range of potential risk factors emerging in the first years of life. The current review has identified several key themes for future work. First, a greater focus on examining longitudinal relations between different markers is important to mapping causal pathways for symptom development. Second, using multiple methods and measures to assess the integrity of neurocognitive systems that could form developmental pathways to ASD is an important strategy. Targeted intervention studies are one way to test the causality of these factors, in addition to their potential for ameliorating symptoms of ASD. Third, using dimensional measures of symptom profile and studying a range of different risk groups will be critical to understanding the specificity of early markers to symptoms of ASD versus other comorbid conditions (such as ADHD), and the generalizability of early markers outside multiplex families. Fourth, greater collaboration between studies in order to increase sample sizes and increase consistency between analysis strategies is important. Increased sample sizes will also allow greater understanding of subgroups, and the use of more complex multifactorial models to understand the relations between different risk factors. Fifth, researchers should examine the reliability and validity of experimental tasks that provide potential markers for ASD in early development, as is the case for standardized behavioral measures. Sixth, the heterogeneity in the domains in which early markers are seen suggests that a focus on domain-general measures of information processing in early development is important. Finally, beginning to map predictors of heterogeneity in both short and long-term developmental course within the infants who are later diagnosed with ASD will be critical to both theoretical accounts of ASD development, and clinical measures derived from such research.

## Figures and Tables

**Table 1:** Early predictors of developmental outcome in high-risk sibling studies.

Reference	*n*	Predictors	Outcome specification	Findings
Bedford et al., 2012	HR-ASD *n* = 12, 4F HR-Atypical *n* = 9, 7F HR-TD *n* = 14, 10F LR *n* = 38, 10F PB: Community clinical diagnosis; DAWBA, SCQ, clinical judgment. Entry: By 6 months. Ex: Diagnosis of medical or neurological condition at entry; ASD in first-degree relatives for LR.	*7* *m (6–10* *m), 13m (11–18* *m)* Gaze-following (eye-tracker task; person looks at one of two objects on the screen. Measures: The direction of the first look and the amount of time spent looking at the referenced object).	*BASIS 36* *m diagnostic* HR-ASD: ICD-10 criteria for ASD, using information from the ADOS, ADI-R and clinical judgment. HR-Atypical: (a) met ADI-R criteria for Autism (*n* = 1), (b) met ADOS criteria for ASD (*n* = 6), (c) <1.5 *SD* below population mean on the MSEL Early Learning Composite or RL AND EL subscales (*n* = 1), or meeting both of points b and c above (*n* = 1). HR-TD: Not in the above two groups.	*7* *m:* No differences related to outcome. *13* *m:* HR-ASD, HR-Atypical <HR-TD and LR in looking time to the referenced object. Reduced looking time was correlated with higher 24 month ADOS-G social-communication algorithm total score (more symptoms) within HR infants.

Chawarska et al., 2013	ASD *n* = 12, 4F (?HR?LR) HR-Atypical *n* = 22, 4F HR-TD *n* = 15, 7F LR-TD *n* = 35, 16F PB: ADI and/or ADOS, clinical judgment. Entry: By 6 months Ex: Gestational age <34 weeks, any hearing or visual impairment, nonfebrile seizure disorders, or known genetic syndrome; LR only – no history of ASD in 1st or 2nd degree relatives.	*6* *m* Videos of women making sandwiches, speaking to the child, looking at toys. Gaze measured with eye-tracking.	*24* *m (32%) or 36* *m (68%)* ASD: ADOS, MSEL, language assessments and clinical judgment. HR-Atypical: any clinically significant atypical features in the 2nd or 3rd years of life (e.g. language or developmental delay, abnormal social-communication or repetitive behaviors), but didn’t meet criteria for ASD. LR-TD, HR-TD: no developmental concerns in 2nd or 3rd years.	*6* *m* ASD < other groups on amount of eye-tracking data; ASD < other groups on percent looking to the face during videos.

Christensen et al., 2010	HR-ASD *n* = 17, 3F (?HR?LR) HR-Other Delays *n* = 12, 6F HR-No Delays *n* = 29, 16F LR-TD *n* = 19, 12F PB: Community clinical diagnosis; confirmed with ADI-R, ADOS, DSM-IV or record review and ADOS where necessary. Entry: By 18 months. Ex: Severe visual, hearing or motor impairment, or medical conditions associated with ASD; LR only – history of ASD in 1st or 2nd degree relatives; gestational age <36 weeks or >42 weeks; abnormalities in pregnancy or neonatal period; chronic health conditions, past hospitalizations or significant injuries for proband or sibling; diagnosed developmental or learning disabilities, or behavioral disorders in the proband. Proband in normal range on SCQ.	*18* *m* 4 min free play (play stove, pot with lid, sponges, play sandwich, brush, cup, plate, spook, fork, square block, cylindrical block, two Ernie dolls). Coded functional (object-directed, self-directed, doll-directed, other-directed), symbolic (substitution play, imaginary play, doll-as-agent play), and repeated play (functional/symbolic repeated acts and non-functional/symbolic repeated acts). Analyzed behavior counts.	*36* *m* HR-ASD: ADOS–met criteria for ASD at 36 m AND 18 and/or 24 m; SCQ scores consistent with ASD or Autism. HR-Other Delays: did not meet criteria for ASD at 18 m, 24 m AND 36 m; AND deficits in general cognition (Mullen composite <78 and one non-language and one language subtest ≤1.5 *SD* below average), language (≤2 *SD* below average on either or ≤1.5 *SD* on both Mullen RL and EL scales); or social behaviors (elevated scores on ADOS social-communication algorithm but did not meet criteria for Autism or ASD) at 36 months; OR parents or examiner noted other concerns about development. HR-No Delays: HR infants who did not fall into the ASD or Other Delays categories. LR-TD: LR infants who did not meet criteria specified for the HR-ASD and HR-Other Delays groups.	*18* *m* HR-ASD < LR-TD on functional play (less self-directed and other-directed). HR-ASD > LR-TD on non-functional repeated play acts. Effects were no longer significant after verbal mental age was covaried.

Clifford et al., 2013	HR-ASD *n* = 17, 6F HR-Atypical *n* = 12, 9F HR-TD *n* = 24, 17F LR *n* = 50, 28F PB/Entry/Ex: see Bedford et al., 2012.	*7* *m (6–9* *m), 14* *m (12–15* *m)* IBQ-R – parent-report temperament questionnaire that measures 17 subscales (Activity Level, Smiling and Laughter, Fear, Distress to Limitations, Duration of Orienting, Soothability, Vocal Reactivity, Approach, Falling Reactivity, High and Low Intensity Pleasure, Perceptual Sensitivity, Sadness, Cuddliness, Social Fear and Attention Shifting); each of the subscales is assigned to one of three overarching domains (Surgency, Negativity and Effortful Control).	*BASIS 36* *m diagnostic*	*7* *m*: HR-ASD show higher levels of Perceptual Sensitivity than HR-TD (with others intermediate). *14* *m*: HR-ASD show less Effortful Control than LR and HR-atypical; less Smiling and Laughter than LR; more Perceptual Sensitivity than HR-TD; less Cuddliness than all other groups.
Cornew et al., 2012	ASD *n* = 9, 1F (1 LR, 8 HR) HR-NS *n* = 30, 17 or 18F LR *n* = 43, 20 or 21F PB: Clinical judgment, ADOS, ADI-R, diagnostic reports. Entry: Date of test. Ex: No family history of ASD	*17–20* *m* Social referencing to parent in response to exciting toy and object approach/withdrawal in response to parent object-directed emotional expression.	*36* *m* DSM-IV based on meeting criteria for ASD on the ADOS, and clinical judgment based on ADOS and ADI-R.	ASD showed longer latency to reference and 6 of the 9 infants failed to reference; only LR showed correct behavioral regulation in response to parent emotional expression.

Damiano et al., 2012	HR-ASD *n* = 8 HR-no ASD *n* = 12 (*n* = 2 LD, 2 DD, 8TD) LR-TD *n* = 20 (2 LD, 18 TD) (HR *n* = 20, 7F) (LR *n* = 20, 6F) PB: ADOS, ADI-R, clinical judgment. Entry: 12–23 months. Ex: Severe sensory or motor impairments; identified genetic or metabolic disorders. LR only: ASD in first-degree relatives.	*12–23* *m* Repetitive and Stereotyped Movement Scales: Based on STAT videos. Code body-related (flapping, stiffening, rubbing, patting) and object-related (spin, rock, roll, collect, swipe, rub, move, line, clutch) RSMs. Analyzed rate and inventory for each category.	*32 (27 to 42* *m)* ASD: Licensed psychologist based on ADOS, ADI-R, MSEL. No other definitions given.	*12–23* *m* No relation between rate of RSMs and later diagnosis. Object > Body Inventory for HR-no ASD and HR-TD, not for HR-ASD.

del Rosario et al., 2013	6 m/12 m/18 m/24 m/36 m HR-ASD *n* = 11/16/10/10/10, 14.3%F HR-Concerns (data not reported) HR-TD *n* = 7/13/15/18/27, 48.5%F PB: ADOS, ADI-R, DSM-IV, clinical judgment. Of note, siblings all had a diagnosis of Autistic Disorder. Entry:? Ex:?	*6* *m, 12* *m, 18* *m, 24* *m, 36* *m* Carey Temperament Scales – parent-report questionnaire that measures nine domains of temperament (Activity, Adaptability, Approach, Mood, Intensity, Distractibility, Persistence, Sensory Reactivity, Rhythmicity). MSEL–standardized assessment of cognitive skills.	*24* *m (n* *=* *7) or 36* *m (n* *=* *37)* HR-ASD: Clinician judgment based on ADOS, SCQ, DSM-IV-TR, MSEL, VABS. HR-Concerns: ADOS within 1 point of ASD cut-off; OR 2 *SD* < mean on one MSEL scale, OR 1.5 *SD* < mean on two or more MSEL scales; OR clinician concern about development. HR-TD: Did not raise concerns, appeared to be developing typically.	*6* *m to 36* *m* Slope of change differed between HR-ASD and HR-TD in Approach (HR-TD flatter than HR-ASD), Adaptability (HR-TD more downward sloping than HR-ASD) and Activity (HR-TD flatter than HR-ASD). *6* *m, 12* *m*: HR-ASD < HR-TD for Adaptability (low score = faster adaptation). *6* *m:* HR-ASD < HR-TD for Approach (low score = higher approach).

Elison et al., 2013	HR-ASD *n* = 16, 5F HR-no ASD *n* = 40, 20F LR *n* = 41, 17F PB: Community clinical diagnosis, and ADI-R. Entry: By 6 months. Ex: Significant medical condition known to affect brain development; sensory impairment; low birth weight (<2200 g) or prematurity (<36 wks); perinatal brain injury secondary to maternal complications or exposure to specific medications or neurotoxins during gestation; non-English speaking family; contraindication for MRI; adoption; first degree relative with idiopathic intellectual disability, psychosis, schizophrenia, or bipolar disorder.	*6* *m* Diffusion tensor imaging, Gap attention task – measures reaction time to saccade to peripheral stimulus from central stimulus when peripheral appears during the central stimulus presentation (overlap), or simultaneous with (baseline), or after (gap) its disappearance. Stimuli were mixed social and nonsocial pictures.	*24* *m* HR-ASD: Met criteria for ASD on the ADOS. HR-no ASD: Did not meet criteria for ASD on the ADOS.	*6* *m:* HR-ASD > HR-no ASD = LR on overlap saccadic RT; HR-ASD >LR with HR-no ASD intermediate on gap saccadic RT. Reaction times in the overlap condition correlated with radial diffusivity in the splenium.

Elsabbagh et al., 2011	Freeze-frame: HR *n* = 22, ?F ERP: HR *n* = 16, ?F PB: Community clinical dx; DAWBA, clinical judgment. Entry: By 9 months. Ex: Prematurity, low birth weight, medical or neurological conditions, sensory or motor problems.	*9* *m* Freeze-frame inhibition of saccade task – central stimulus is repetitive and simple (‘boring’) or trial-unique and complex (‘interesting’); infants must inhibit saccades to peripheral distractors. ERPs to direct and averted gaze Amplitude and latency of P1, N290, P400.	*36* *m* Correlations with ADOS scores.	*9* *m*: Lower proportion of looks to boring distractors predicted more impairment in social interaction.
Elsabbagh et al., 2012	HR-ASD *n* = 16, ?F (9 of which in early and persistent group) HR-no ASD *n* = 33, ?F LR *n* = 45, ?F Full initial group: (HR *n* = 54, 34F) (LR *n* = 50, 29F) PB/Entry/Ex: see Bedford et al., 2012.	*6–10* *m* Dynamic direct versus averted gaze ERP. Gaze shifts toward and away from infant; amplitude and latency of P1, N290, P400.	*BASIS 36* *m diagnostic*	*6–10* *m:* HR-ASD showed no significant P400 difference between gaze shifts away and toward the infant, unlike HR-no ASD and LR.

Elsabbagh et al., 2013c	HR-ASD *n* = 17, 6F HR-Atypical *n* = 12, 9F HR-TD *n* = 24, 17F LR *n* = 50, 28F PB/Entry/Ex: see Bedford et al., 2012.	*7* *m (6–10* *m), 14 (12–15* *m)* Face pop-out (arrays of 5 objects, one of which is a face and another one is a frequency scrambled face); eye-tracking. Assess first gaze to face (face pop-out); total looking time to face (face looking) and number of interest areas viewed (visual foraging).	*BASIS 36* *m diagnostic*	*7* *m*: No differences based on outcome. *14* *m:* No differences based on outcome.

Elsabbagh et al., 2013a	HR-ASD *n* = 17, 6F HR-Atypical *n* = 12, 9F HR-TD *n* = 24, 17F LR *n* = 48, 28F PB/Entry/Ex: see Bedford et al., 2012.	*7* *m (6–10* *m), 14* *m (12–15* *m)* Eye to mouth index during movies with single or multiple features moving (e.g. eyes, mouth, hands).	*BASIS 36* *m diagnostic*	*7* *m, 14* *m*: No outcome group differences in modulation of looking to eyes and mouth by movement type.

Elsabbagh et al., 2013b	HR-ASD *n* = 17, 6F HR-Atypical *n* = 12, 9F HR-TD *n* = 24, 17F LR *n* = 50, 28F PB/Entry/Ex: see Bedford et al., 2012.	*7* *m (6–10* *m), 14* *m (12–15* *m)* GAP attention task–measures reaction time to saccade to peripheral stimulus from central stimulus when peripheral appears during the central stimulus presentation (overlap), or simultaneous with (baseline), or after (gap) its disappearance.	*BASIS 36* *m diagnostic*	*7* *m*: No significant difference related to outcome. *14* *m*: HR-ASD showed prolonged overlap-baseline RT (disengagement) relative to other groups. No consistent increase in speed of disengagement between 7 and 14 months in HR-ASD and HR-Other, unlike other groups.

Feldman et al., 2012	HR-ASD *n* = 9, 3F Sib-no ASD *n* = 99, c. 31F PB: ADI-R, diagnostic reports Entry: 1–24 m Ex: known biological, birth or medical conditions associated with DD; no older sib with known genetic syndromes related to ASD.	*1–24* *m* Parent Observation of Early Markers Scale (POEMS); Parent-report measure of problems in socio-communicative development, restricted interests, repetitive behaviors, behavioral problems, emotional and other problems (e.g. waiting, transitions, novelty, sensory issues, regulatory control, attention, tracking, motor control).	*36* *m* Community diagnosis confirmed with ADI-R in three cases.	*3–24* *m:* Higher total POEMS scores and elevated POEMS items (scores = 3 or 4) in HR-ASD. Discriminating items were: *9* *m:* Interest in faces, shifts attention to person, mood, response to name and waiting; *12* *m:* Interest in faces, waiting, shifts attention to person, imitates sounds or words. *18* *m*: Waiting, imitates sounds or words, coordinates point and gaze.

Flanagan et al., 2012	*Sample 1:* HR-ASD *n* = 10, 0F HR-Atypical *n* = 13, 5F HR-TD *n* = 17, 12F *Sample 2 (no outcome):* HR *n* = 20, 11F LR *n* = 20, 13F PB: ADOS, ADI-R, clinical judgment. Entry: by 6 months. Note: 7/9 parents who filled out parent concern questionnaire in HR-ASD group expressed concern at study entry. Ex: child's primary language exposure other than English, low birth weight (<2250 g), premature birth (<37 weeks), severe birth trauma, head injury, prenatal illicit drug or excessive alcohol exposure, or severe birth defects.	*6* *m* Head lag during pull-to-sit in MSEL; scored by trained examiner as binary variable (present, absent).	*36* *m* HR-ASD: Met ADOS criteria for ASD and had clinical judgment of ASD. HR-Atypical: ≥1.5 *SD* below mean on MSEL RL or EL, met ADOS criteria for ASD on the communication or reciprocal social interaction domains with clinical judgment of social/communication delay, OR met criteria for both domains without clinical judgment of delay. HR-TD: Did not meet criteria for HR-ASD or HR-Atypical.	*6* *m:* HR-ASD significantly more likely to show head lag compared to other groups (9/10 HR-ASD; 7/13 HR-Atypical and 6/17 HR-TD; 15/20 HR and 7/20 LR showed head lag).

Garon et al., 2009	HR-ASD *n* = 34, 12F HR-no ASD *n* = 104, 53F LR *n* = 73, 38F PB: Expert clinician judgment based on DSM-IV, with ADOS and ADI-R in most cases. Entry: 84%HR and 80%LR by 6 months, remainder by 12 months. Ex: No known genetic syndrome related to ASD; <37 weeks gestation). LR only: no 1st or 2nd degree relative with ASD.	*24* *m* Toddler Behavior Assessment Questionnaire Revised (TBAQ-R) – parent-report measure of temperament; subscales are Positive Anticipation, Attention Shifting, Activity Level, Inhibitory Control, Attention Shifting, Attention Focus, Low Pleasure, High Pleasure, Social Fear, Anger, Soothability. Analyzed with discriminant function analysis.	*36* *m* HR-ASD: Combination of ADI-R, ADOS, DSM-IV. HR-no ASD: Didn’t meet criteria for ASD.	*24* *m:* HR-ASD < HR-no ASD and LR on Behavioral Approach (Positive Anticipation–particularly excitement over cues for a reward, Attention Shifting–particularly ease of capturing attention with social cues, and Activity Level–particularly motor activity with no specific goal), even after controlling for IQ (MSEL), ASD symptoms (ADOS) and sex. Behavioral approach also predicted variance in ADOS social affective symptoms at 36 m.

Hudry et al., 2013	HR-ASD *n* = 17, 6F HR-Atypical *n* = 12, 9F HR-TD *n* = 24, 17F LR *n* = 48, 28F PB/Entry/Ex: see Bedford et al., 2012.	*6–9* *m; 12–14* *m* CDI – parent report measure of language skills in receptive and expressive domains. VABS–Receptive and Expressive Language scales from parent report measure of adaptive functioning; MSEL RL and EL AE – Receptive and Expressive Language subscales of cognitive assessment. Receptive-Expressive used as index for all measures.	*BASIS 36* *m diagnostic*	*6–9* *m*: No group differences. *12–14* *m*: HR versus LR less receptive advantage on CDI, though not related to outcome at this age; no group differences on MSEL, VABS.

Hutman et al., 2010	*12/18* *m* HR-ASD *n* = 12/14, 64%F (13HR 1LR) HR-no ASD *n* = 74/86, 50%F LR *n* = 52/47, 56%F PB: ADI-R, ADOS, DSM-IV. Entry: 6, 12 or 18 months. Ex: For LR, history of ASD or DD in extended family.	*12, 18* *m* Response to Distress (examiner pretends to hit finger with mallet during game with xylophone). Code behavior and facial expression.	*36* *m* HR-ASD: Clinical diagnosis based on ADOS, ADI, DSM-IV.	*12* *m, 18* *m*: HR-ASD showed lower levels of attention and affective response to distress than other groups.

Hutman et al., 2012	*12* *m* ASD *n* = 15, 4F (14HR 1LR) Other *n* = 12, 5F (8HR 4LR) HR-TD *n* = 59, 33F LR-TD *n* = 43, 18F PB: Clinician judgment based on direct observation and parent report. Entry: By 12 months. Ex: For LR, no family history of neurodevelopmental disorders.	*12* *m* Play with examiner, and response to distress (examiner pretends to hit finger with mallet during game with xylophone). Coded attention direction and attention shifts.	*36* *m* ASD: Clinical diagnosis based on ADOS, ADI, DSM-IV. HR-TD/LR-TD: No single MSEL scale score >2 *SD* below mean, no more than one >1.5 *SD* below mean; summed social-communication algorithm from ADOS more than one point below ASD cut-off. Other: Didn’t fall into the other categories.	*12* *m*: ASD and Other show less attention to social targets during distress condition but not play condition than HR-TD and LR-TD. Number of looks to social targets was greater in distress versus play condition for all groups except ASD. No significant effects for non-social targets.

Iverson and Wozniak, 2007	HR-ASD: *n* = 2, 0F HR-no ASD *n* = 19, 15F LR *n* = 18, 10F. PB: Community clinical diagnosis, met criteria for Autism on the ADOS. Entry: By 6 months. Ex: Prematurity, complications in pregnancy, not monolingual English household.	*5m–14* *m (monthly)* CDI – parent report measure of language skills in receptive and expressive domains. Coding of milestone onsets (sitting, reduplicated babble, showing, pointing, walking, first word); rhythmic limb movements; posture bouts.	*18* *m* ASD: Scored above cut-off on PDDST-II, and received subsequent community diagnosis.	*5m-14* *m* One child never produced reduplicated babble; one child delayed in babble onset (10 m) and showed an increase in the proportionate production of finger rhythmicities at babble onset; both showed delayed onset of walking (15, 16 m), pointing (14, 13 m) and first words (18 m).

Landa and Garrett-Mayer, 2006	ASD *n* = 24, ?F (22HR 2LR) LD *n* = 11, ?F (9HR 2LR) UA *n* = 52, ?F (29HR, 23LR) (HR *n* = 60, 25F) (LR *n* = 27, 10F) PB: ADOS, ADI-R. Entry: By 14 months. Ex: English not first language; low birth weight; severe birth trauma; head injury; prenatal drug use or excess alcohol exposure; severe birth defects.	*6* *m (5–10* *m), 14* *m (13–17* *m)* MSEL – standardized assessment of cognitive skill.	*24* *m* ASD: Met ADOS criteria for ASD plus clinical judgment. LD: ≥1 *SD* below mean for RL/EL on PLS or CDI OR met ASD criteria on only communication domain of ADOS; AND judged to have language delay. UA: Didn’t meet criteria for ASD or LD.	*6* *m*: FM: LD< UA. *14* *m*: FM, RL: LD < UA, ASD < UA. EL, GM: ASD < UA but not LD. TD: VR = RL; ASD, LD: VR> RL.

Landa et al., 2007	HR-ASD *n* = 30, 5F Early-ASD *n* = 16, 2F Late-ASD *n* = 14, 3F HR-BAP *n* = 19, 6F HR-non BAP *n* = 58, 37F LR *n* = 18, 7F PB/Entry/Ex: see Landa and Garrett-Mayer (2006).	*14* *m* CSBS DP – assessment of social communication behaviors.	*Later of 36 (n* *=* *88) or 30* *m (n* *=* *19)* HR-ASD: Met ADOS criteria for ASD, DSM-IV and clinical judgment. Early-ASD if had clinical impression of ASD at 14 m, Late-ASD if not. HR-BAP: MSEL EL or RL ≤1.25 *SD* below mean OR ADOS reciprocal social interaction algorithm met criteria for ASD AND examiner considered there was impairment in social behavioral or communication skills. HR-non BAP: Test scores within normal limits.	*14* *m:* Early-ASD worse than all other groups on IJA, Behavior Regulatory Bids, Inventory of Gestures and Consonants in Syllables, and Action Schema Inventory. Worse than all groups except Late-ASD for Gaze Shifts. Worse than all except LR for Shared Positive Affect and Action Schema toward others. RJA and Word Inventory differed from non-BAP but not other groups. Action Schema Inventory differed from all except Late-ASD. Late-ASD produced fewer Gaze Shifts than non-BAP group.

Leonard et al., 2013	HR-ASD *n* = 17, 6F HR-Atypical *n* = 12, 9F HR-TD *n* = 24, 17F LR *n* = 50, 28F PB/Entry/Ex: see Bedford et al., 2012.	*7* *m (6–10* *m), 14* *m (12–15* *m)* MSEL GM and FM–motor scales of standardized assessment of cognitive skill. VABS–motor scales of parent-report measure of adaptive functioning (as a questionnaire).	*BASIS 36* *m diagnostic*	*7* *m, 14* *m:* No significant differences related to outcome on motor measures.

Loh et al., 2007	HR-ASD *n* = 8, 5F HR-no ASD *n* =9, 6F LR *n* = 15, 5F PB: Community clinical diagnosis, ADOS, ADI-R, clinical judgment. Entry: most pre-6 months. Ex: LR only – birth weight <2500 g, prematurity, 1st or 2nd degree relatives with ASD.	*12, 18* *m* (visit within 4 weeks) Repetitive Behaviors: Coded during AOSI. Thelen's taxonomy of 47 repetitive behaviors (movement of part of body that is repeated in same form at least twice at regular short intervals of 1s or less). Posturing–atypical/non-functional position, held for at least .25 s. 9 behaviors at 12 m, 9 at 18 m had high enough ICC.	*36* *m* HR-ASD: Clinical diagnosis based on DSM-IV using ADOS, ADI, cognitive, language and adaptive measures. Research diagnosis based on ADI-R (met criteria on social algorithm and either communication or behavior), ADOS, DSM-IV.	*12* *m*: Arm wave HR-ASD = HR-no ASD > LR. *18* *m*: Arm wave HR-ASD > LR, HR-no ASD (but only when use clinical diagnosis; not significantly different using research diagnosis). Hands to ears HR-ASD, HR-no ASD> LR.

Macari et al., 2012	ASD *n* = 13, 3F (12HR 1LR) TD *n* = 34, 24F (12HR 22LR) Atypical *n* = 37, 7F (29HR 8LR) PB/Entry/Ex: see Chawarska et al., 2013.	*12* *m* ADOS-Toddler item analysis. Semi-standardized play-based assessment of ASD symptoms; used classification trees.	*24* *m* ASD: Exhibited marked delays and abnormalities in social interaction and communication skills, evident across instruments and contexts and in conjunction with parent report of similar atypical behavior patterns. TD: No developmental concerns at 18 or 24 months. Atypical: Any atypical features at 18 or 24 months; >1.5 *SD* below mean on any MSEL subscale or exhibited behavior problems (*n* = 20); had socio-communicative delays, atypical language or sensory and repetitive behaviors (*n* = 17).	*12* *m* Showing, Overactivity and Initiating Joint Attention predicted TD versus non-TD (ASD, Atypical); PPV 0.7; NPV 0.82; Se 0.71; Sp 0.86. Level of Engagement, amount of Requesting, Imitation, Fussiness, Showing, Gestures and Intonation distinguished ASD from non-ASD groups. PPV 0.79; NPV 0.97; Se 0.85; Sp 0.96. Four subgroups: ASD1–low Engagement (*n* = 5); ASD2 – poor Imitation, Showing and Requesting; low Fussiness, good Gestures and Engagement (*n* = 4); ASD3 – same as ASD2 but poor Gestures (*n* = 2); ASD4 = good Engagement, poor Requesting and Imitation, Fussy, unusual Vocalizations (*n* = 2).

Mitchell et al., 2006	HR-ASD *n* = 15, 5F HR-no ASD *n* = 82, 41F LR *n* = 49, 22F PB: Community clinical, ADOS, clinical judgment. Entry: Most by 6 months? Ex: No specific neurological or genetic conditions accounting for ASD diagnosis in proband, absence of significant motor and sensory impairment; LR only – birth weight <2500 g, prematurity, 1st or 2nd degree relatives with ASD.	*12* *m (11 to 15* *m), 18* *m (17 to 21* *m)* CDI W + G – Parent-report measure of child language development (Phrases Understood, Vocabulary Comprehension, Vocabulary Production, Early Gestures (e.g. first communicative gestures), Late Gestures (e.g. pretending to be a parent)).	*24* *m* ASD: Based on ADOS plus DSM-IV (Se and Sp of 70% and 98.4% at 3 years).	*12* *m*: HR-ASD < HR-no ASD, LR on Phrases Understood, Late Gestures, Early Gestures. *18* *m*: HR-ASD < HR-no ASD, LR on all measures. HR-no ASD < LR for Late Gestures.

Nadig et al., 2007	ASD *n* =10, ?F LD *n* = ? Other *n* = ? TD *n* = ? *6* *m* (HR *n* = 55, 21F) (LR *n* = 43, 20F) *12* *m* (HR *n* = 101, 45F) (LR *n* = 46, 28F) PB: ADOS, SCQ (SCQ for TD also). Entry: 1, 3, 6, 12 (and 18 m). Ex: No info.	*12* *m* Response to name: Experimenter calls child's name whilst playing with a toy, from behind them. Pause for 3 s and repeat up to 3 times. Scored as response if child makes eye contact (1 point for first call, 2 points for second, 4 for no response after third etc.).	*24* *m* ASD: Met criteria for ASD on the ADOS, and DSM-IV. LD: MSEL EL <1 *SD* and didn’t meet criteria for ASD on the ADOS. Other: Atypical development.	*12* *m:* Se for failure to respond to name was 0.5 for ASD, 0.39 for any DD (of *n* = 10 with ASD, 3 failed to orient at all). Sp (of 71 children with data at 12 and 24 months) was 0.89 for ASD, 0.94 for any DD.

Ozonoff et al., 2008a	ASD *n* = 9, 0F (8HR 1LR) Other Delays *n* = 10, 3F (6HR 4LR) TD *n* = 47, 25 F (21HR 26LR) PB: Unclear. Entry: Unclear. Ex: No info.	*12* *m* Object manipulation: Children manipulated round metal lid, round plastic ring, rattle, plastic baby bottle (30 s each). Coded for ‘typical’ (shaking, banging, mouthing, throwing) and atypical (spinning, rolling, rotating, unusual visual exploration).	*24, 36* *m* ASD: Met criteria for ASD on ADOS and SCQ, and clinical best estimate based on DSM-IV was consistent. Other Delays: >1.5 *SD* MSEL, or clinical best estimate of behavior problem or developmental delay and did not meet criteria for ASD. TD: Did not meet criteria for ASD or Other.	*12* *m: n* = 7 (77.8%) of the ASD group showed at least one atypical object exploration that was >2 *SD* above TD group mean, versus *n* = 5 (50%) Other Delays and *n* = 11 (23.4%) TD. Unusual visual exploration was most common behavior. Within children with data at 36 months, higher frequency of spinning objects predicted higher 36 month ADOS RB and COM + SOC; longer duration of unusual visual exploration predicted higher ADOS scores and lower MSEL scores.

Ozonoff et al., 2010	ASD *n* = 25, 6F (19HR 3LR) TD *n* = 25, 5F (25LR) PB: Meet criteria for ASD on ADOS, SCQ (SCQ also for TD). Entry: 6 months? Ex: Gestational age < 36 weeks; known genetic disorder in older sibling. For TD: any developmental, learning or medical condition in any older sib; ASD in up to 3rd degree relative.	*6, 12, 18* *m* Social behaviors coded during MSEL VR: Gaze to faces, gaze to objects, smiles, nonverbal vocalizations, single word and phrase verbalizations; social smiles directed vocalizations. Examiner rated social engagement (rare, occasional, frequent): frequency of eye contact, shared affect and overall social responsiveness. Summed to give social engagement composite score.	*36* *m* ASD: Met criteria for ASD on ADOS, and clinical best estimate based on DSM-IV was consistent. TD: MSEL composite > 78, no more than one subtest ≤1.5 *SD* below mean AND no MSEL test > 2 *SD* below mean AND two or more points below ASD cutoff of ADOS AND clinical judgment of no DD .	*6* *m*: No significant group differences, with non-significant trend for ASD to show more social behaviors. *12* *m*: gaze to faces and social smiling ASD <TD. *18* *m*: eye contact, social smiling, social responsiveness ASD< TD. No group differences in object variables. *12–24* *m*: ASD group show decline in all behaviors such that significant differences emerge. 83% of parents did not report a loss in this period.

Paul et al., 2010	ASD/BAP *n* = 14, ?F (13HR 1LR) HR-no ASD *n* = 11, ?F LR-no ASD *n* = 20, ?F PB: Community clinical, ADI-R; Entry: By 12 months? Ex: Gestational age <32 weeks; known sensory deficits, known genetic syndromes, known neurological or significant chronic medical disorder. LR only-any siblings with diagnosis of ASD or any developmental disorder.	*6* *m, 9* *m, 12* *m* Vocal samples from parent-child interaction. MSEL–standardized assessment of cognitive skill.	*24* *m* ASD: Clinical diagnosis based on ADOS-T (at least 5 points), MSEL (verbal < nonverbal, RL < EL), and observation of social atypicalities. BAP: >8 on ADOS-T, less pronounced social atypicalities, not MSEL pattern stated for ASD above, not just language delay. HR-no ASD: ADOS-T 9 or below.	*6* *m*: Number of middle consonant types was smaller in ASD/BAP; MSEL EL scores were lower in ASD/BAP. *9* *m*: Number of late consonant types was smaller in ASD/BAP. *12* *m*: Total number of different consonant types was lower in ASD/BAP. (i.e. newly emerging behaviors predict diagnosis at all ages).

Rozga et al., 2011	ASD *n* = 17, 3F (15HR 2LR) HR-no ASD *n* = 84, 47F LR *n* = 66, 30F (numbers for individual tasks are lower) PB: ADOS, ADI-R, DSM; SCQ in TD. Entry: By 6, 12, 18 months. Ex: Gestational age <36 weeks; known genetic disorder in older sibling; English as second language. LR only: any developmental, learning or medical condition in any older sibling; no ASD in extended family.	*6* *m, 12* *m* *6* *m:* Free play mother-infant interaction (coded gaze to mother's face, smile, non-distress vocal, social smile, social vocal); still face (coded gaze direction and affect). *12* *m*: ESCS–structured assessment of social communication behaviors.	*Later of 24* *m/36* *m* ASD: Met criteria for ASD on ADOS, DSM-IV and clinical judgment.	*6* *m:* No significant differences. *12* *m*: HR-ASD show less high-level IJA, Requesting, RJA than other groups.

Sheinkopf et al., 2012	HR-ASD: *n* = 3, ?F LR *n* = 18, 10F PB: Community clinical diagnosis of ASD, over threshold for “autism” on the ADOS. Entry: By 6 months. Ex: Prematurity, complications in pregnancy, not monolingual English household.	*6* *m* Analysis of acoustics of first utterances of cry episodes from audio-video recordings in infants’ home.	*36* *m* HR-ASD: Clinical judgment based on DSM-IV using ADOS, met ADOS criteria for ASD.	*6* *m*: One HR-ASD had second highest pitch in the sample; second HR-ASD had third highest pitch range in the sample. Both had cries with lowest average phonation in both pain and non-pain related groups of cries.

Shen et al., 2013	HR-ASD *n* = 10, 2F DD *n* = 11, ?F (8HR) TD *n* = 34, ?F (15HR) HR *n* = 33, 11F; LR *n* = 22, 7F PB/Entry/Ex: see Ozonoff et al., 2010.	*6–9* *m, 12–15* *m* 3 T scan during natural sleep; T1 and T2-weighted. Measures were cerebral volume and volume of extra-axial fluid.	*24* *m (n* = *17) or 36* *m (n* = *38)* HR-ASD: Clinical judgment based on DSM-IV using ADOS and clinical observation. DD: Not HR-ASD or TD. TD: ADOS score <4 on Mod 1 or <5 on Mod 2; AND overall MSEL score of ≥85; AND not more than one MSEL score ≤35.	*6–9* *m, 12–15* *m*: Rate of growth of total cerebral volume higher in HR-ASD than TD or DD; significantly larger (7%) by 12–15 m. HR-ASD had significantly greater total extra-axial fluid than other groups (controlling for head size) at 6–9 m (20% more) and 12–15 m (33% more). Greater extra-axial fluid volume during infancy was predictive of higher ADOS SOC + COM scores at 24–36 months, with longer degree of elevation predicting worse COM scores.

Stone et al., 2008	ASD *n* = 19, ?F (9HR) Non-ASD: DD *n* = 6, ?F (4HR) LI = 1, ?F (1HR) BAP *n* = 8, ?F (8HR) No concerns *n* = 37, ?F (37HR) (HR *n* = 59, 25F) (Note: groups are a mixture of high-risk sibs and clinically referred children). PB: Children with ASD. Entry: by 24 months. Ex: Severe sensory or motor impairments; identified genetic or metabolic disorders.	*12–23* *m* Screening Tool for Autism in Two-Year-Olds: Set of 12 activities for eliciting social and communicative behaviors.	*24* *m* ASD: Clinical judgment based on ADOS, DSM-IV. DD: MSEL ELC and FM or VR ≤1.5 *SD* below mean. LI: no DD and RL and/or EL ≤1.5 *SD* below mean. BAP: Didn’t meet criteria for groups above AND clinical socio/communicative concerns AND met criteria for ASD on the ADOS reciprocal social interaction algorithm. No concerns: Didn’t fall into groups above.	*12–13* *m:* Poor performance (lots of false positives). *14–24* *m:* With a cut-score of 2.75, Se 0.93, Sp 0.83, PPV 0.68, NPV 0.97.

Sullivan et al., 2007	HR-ASD *n* = 16, 2F HR-BAP *n* = 8, 1F HR-Non BAP *n* = 27, 15F PB: ADOS, ADI-R, clinical judgment. Entry: By 14 months (54% at 6 m and 100% at 14 m reported concerns in HR group); 7 of 16 HR-ASD entered at 6 months. Note: 7/9 parents who filled out parent concern questionnaire in HR-ASD group expressed concern at study entry. Ex: Child's primary language exposure other than English, low birth weight (<2500 g), premature birth (<35 weeks), severe birth trauma, head injury, prenatal illicit drug or excessive alcohol exposure, known genetic disorder that would confer increased risk for ASD (e.g. fragile X), or severe birth defects.	*14* *m* CSBS DP – assessment of social communication behaviors; response to Look and Point joint attention cue. Response to Joint Attention–Butterworth task. Response to Look Only joint attention cue: Gaze shift plus head turn cue. ADOS–Response to Joint Attention (RJA) items.	*Later of 36 or 30* *m* HR-ASD: Met criteria for ASD on the ADOS and clinical judgment based on DSM-IV. HR-BAP: MSEL or PLS EL or RL ≤1.25 *SD* below mean (*n* = 3) OR met ADOS criteria on the reciprocal social interaction subscale for ASD (*n* = 5) AND examiner considered there was impairment in social, behavioral or communication skills.	*14* *m:* No group differences on Look Only or Look and Point. HR-ASD/HR-BAP < HR-non-BAP for Look Only. Categorical RJA responding predicts diagnosis if group into ‘poor’ RJA responders (pass ≤50% trials) and ‘good’ (pass ≥75% trials); trials = Look Only and Look and Point.

Talbott et al., 2013	HR-ASD *n* = 9 HR-no ASD *n* = 41 LR *n* = 27 PB: Community clinical diagnosis plus SCQ ≥15 (*n* = 29), met ADOS criteria for ASD (*n* = 6) or expert community diagnosis (*n* = 12). LR probands: <12 on the SCQ, with no first-degree relatives with ASD or another neurodevelopmental disorder. Entry:? Ex: Prematurity, extended stays in the NICU, maternal drug or alcohol use during pregnancy, family history of genetic disorders associated with ASD, primary language not English.	*12* *m* CSBS DP – assessment of social communication behaviors. Mother-child interaction. Infant gesture scored from both contexts. Scores are Tokens (total number of gestures produced) and Types (number of meanings conveyed through gesture). Maternal gesture coded as Deictic (only scored If infant looked at referent), Representational and Conventional (only scored if infant looked toward mother). *18* *m* MSEL–standardized assessment of cognitive skills; sum of Expressive and Receptive Language scales.	*Latest of 18* *m, 24* *m or 36* *m* HR-ASD (*n* = 1 18 m, *n* = 2 24 m, *n* = 6 36 m): met ADOS criteria for ASD; clinical judgment. HR-no ASD: no clinical judgment of ASD (4 met ADOS criteria at 24 but not 36 m and were judged to have no ASD).	*12* *m* Lower variety of gestures used (when summed across both contexts) in HR-ASD than HR-no ASD and LR. Variability in infant gesture score was related with language at 18 m within the ASD group only. Maternal gesture was correlated with 18 m language skills in LR and HR-no ASD, but not HR-ASD.

Wan et al., 2012a	HR-ASD 6 m: *n* = 14, 4F; 12 m *n* = 12, 4F HR-no ASD 6 m: *n* = 31, 21F; 12 m *n* = 31, 22F LR 6 m *n* = 47, 29F; 12 m *n* = 48, 31F PB/Entry/Ex: see Bedford et al., 2012.	*6* *m (6–10* *m), 12* *m (12–15* *m)* AOSI, Parent–Child-Interaction (infant measures: Attentiveness to parent, Positive Affect, Liveliness; parent measures: Sensitive Responsiveness, Non-Directiveness; interaction: Mutuality, Engagement Intensity)	*BASIS 36* *m diagnostic*	*6* *m*: No outcome group differences. *12* *m:* HR-ASD > LR on Sensitive Responsiveness and Non-Directiveness; HR-ASD < LR, HR-no ASD on Positive Affect and Attentiveness to parent, Mutuality and Intensity of Engagement. Mutuality, infant Positive Affect and infant Attentiveness to parent predicted outcome independent of age and AOSI scores.

Wolff et al., 2012	HR-ASD *n* = 12, 4F HR-no ASD *n* = 20, 15F PB/Entry/Ex: see Elison et al., 2013.	*6* *m, 12* *m, 24* *m* Fractional Anisotropy (FA) from structural diffusion tensor imaging scans.	*24* *m* HR-ASD: Met criteria for ASD on the ADOS. HR-no ASD: Did not meet criteria for ASD on the ADOS.	*6* *m*: HR-ASD > HR-no ASD FA left fornix, left inferior longitudinal fasciculus, left uncinate, corpus callosum, right posterior limb of internal capsule. *12* *m:* HR-no ASD > HR-ASD left anterior thalamic radiation. *24* *m*: HR-no ASD > HR ASD left anterior limb internal capsule. *6 to 24* *m change:* Rate of change higher in HR-no ASD in bilateral limbic and association fiber tracts; corpus callosum body; let anterior thalamic radiation and all internal capsule divisions.

Yoder et al., 2009	HR-ASD *n* = 6, ?F HR-TD *n* = 34, ?F (HR *n* = 43, 24F LR *n* = 24, 17F) PB: ADOS, ADI-R, clinical judgment. Entry: 12–23 months. Ex: Severe sensory or motor impairments; identified genetic or metabolic disorders; English not primary language. LR only: ASD in first-degree relatives.	*15* *m, 18* *m, 21* *m, 24* *m* Responding to Joint Attention (RJA) – live presentation Weighted Triadic Communication (WTC) – measures the frequency and conventionality of triadic communication (more complex use of language during triadic reference is weighted more highly).	*36* *m* HR-ASD: Clinical diagnosis based on ADOS, ADI-R. SBC (social behavioral checklist) used as outcome social measure.	*15* *m* In HR group, initial RJA predicted later impairment in RJA. *15–24* *m* WTC growth plus initial RJA plus language age predicted parent report measures of social impairment (SBC).

Young et al., 2009	ASD *n* = 3, ?F (2HR 1LR); LD *n* = 5, ?F (5HR 0LR) Other: *n* = 7, ?F (4HR 3LR) TD *n* = 34, ?F (15HR 19LR) (HR *n* = 33, 16F) (LR *n* = 25, 10F) PB: Unclear. Entry: 6 months Ex: No information.	*6* *m* Video-linked face-to-face social interaction (similar to still face paradigm): Coded gaze aversion, negative affect, smiling. Gaze to eye region/gaze to eye and mouth region during each phase of still face (eye mouth index). Gaze to eyes and mouth versus all face regions (inner outer face index).	*24* *m* ASD: DSM-IV, using ADOS, M-CHAT, MSEL, behavioral observation. LD: MSEL EL >1.5 *SD* below mean. Other Concerns: Clinical judgment of global developmental delay, marked shyness, behavior problems like oppositionality or hyperactivity. No Concerns: Did not meet criteria for other groups.	*6* *m:* ASD showed typical patterns of face scanning/smiling behavior. Across groups, more gaze to mouth relates to significantly faster language development (higher EL on MSEL, VABS and CDI at 24 m), and increased rates of growth and 24 m score for VABS socialization.

Young et al., 2011	ASD *n* = 24, 3F (21HR 3LR) HR-Other *n* = 43, 17F HR-TD *n* = 90, 53F LR-TD *n* = 75, 33F PB: Medical record review, supplemented with ADOS and SCQ where necessary. Entry: By 18 m (64.6% by 6 m; 86.2% by 12 m). Ex: For TD: any developmental, learning or medical condition in any older sib; no ASD in extended family.	*12–24* *m* Imitation battery of 10 items; longitudinal Rasch analysis.	*36* *m* ASD: Met criteria for ASD on the ADOS, and clinical diagnosis based on DSM-IV. Other: MSEL composite <78 OR >1 subtest ≥1.5 *SD* below mean OR ADOS social-communication total within 3 points of ASD cut-off and doesn’t meet DSM-IV for ASD. TD: No single MSEL scale score ≥2 *SD* below mean, no more than one ≥1.5 *SD* below mean; MSEL composite >78; ADOS SOC + COM total at least four points below ASD cut-off and does not meet DSM-IV for any DD or ASD.	*12–24* *m:* ASD and HR-Other showed poorer imitation relative to LR-TD across all time-points.

Zwaigenbaum et al., 2005	AOSI/Orienting/IBQ *6* *m* HR-Autism *n* = 4/3/1, ?F HR-ASD *n* = 8/3/5, ?F HR-Non ASD *n* = 32/19/22, ?F LR: *n* = 15/25/12, ?F AOSI/Orienting/IBQ/MSEL/CDI *12* *m* HR-Autism *n* = 7/4/4/5/4, ?F HR-ASD *n* = 12/6/6/6/3, ?F HR-Non ASD *n* = 46/17/29/29/29, ?F LR: *n* = 23/0/19/12/15, ?F PB: community clinical, ADOS, clinical judgment Entry: Most by 6 months. Ex: LR only – birth weight <2500 g, prematurity, 1st or 2nd degree relatives with ASD.	*6–7* *m, 12–14* *m* AOSI – semi-structured observation of autism-like behaviors. Visual Orienting–measures reaction time to saccade to peripheral stimulus from central stimulus when peripheral appears during (overlap) or after (baseline) central stimulus. IBQ – parent report measure of temperament; scales used were Activity Level, Smile and Laugh, Fear, Distress to Limitations, Soothability, Duration of Orienting. CDI Words and Gestures – parent-report measure of child language. MSEL – standardized assessment of cognitive skills.	*24* *m* HR-Autism: over threshold for Autism on ADOS; confirmed clinically. HR-ASD: above threshold for ASD on ADOS; clinical outcomes unclear.	*6* *m*: AOSI: No group differences. Visual Orienting: No group differences. IBQ: HR-Autism show lower activity level than other groups. *12* *m*: AOSI: >6 risk markers 84% Se and 98% Sp for HR-Autism versus HR-non ASD versus LR (significant individual predictors include atypical Eye Contact, Visual Tracking, Disengagement of visual attention, Orienting to Name, Imitation, Social Smiling, Reactivity, Social Interest and Sensory-oriented behaviors). Visual Orienting: Slowing in disengagement between 6 and 12 associated with scores above ASD cut-off on ADOS; correlation between prolonged disengagement and higher 24 m ADOS algorithm scores. IBQ: HR-Autism show more frequent and intense distress, longer duration of orienting to objects than other groups. MSEL: HR-Autism showed lower RL than other groups (EL trend in same direction). CDI: HR-Autism show fewer gestures, understood phrases than other groups.

*Key*: This table includes manuscripts concerning early developmental predictors (pre 24 months) of later outcome (24–36 months) in infants with older siblings with ASD published up until June 2013 and indexed in major search engines (PubMed, Google Scholar, Web of Science). Where authors were aware of manuscripts that have been submitted or are in press, these were also included.

**Table:** 

HR-ASD, High Risk infants who go on to have ASD (per the operationalization in each paper)
HR- no ASD, High Risk infants who do not go on to a diagnosis of ASD
HR-BAP/ATYP/Other, High Risk infants who have ‘atypical’ or broader phenotype outcomes (per the operationalization in each paper)
HR-TD, High Risk infants considered Typically Developing at the outcome time-point
HR, High Risk (infants with older siblings with ASD)
LR, Low Risk (infants without older siblings with ASD)
ASD/TD/Other, collapsing across high-risk and low-risk groups, infants who had outcomes as described above

PB, Proband (characterization of inclusion criteria applied to the ASD diagnosis of the older sibling of the studied infant)
Ex, Exclusion criteria used for the study

PPV, Positive Predictive Value
NPV, Negative Predictive Value
Se, sensitivity
Sp, specificity

m, months

AOSI, Autism Observational Scale for Infants (Bryson et al., 2007a,b)
ADOS, Autism Diagnostic Observational Scale (Lord et al., 2000)
RB, Restrictive and Repetitive Behaviors total score
SOC, Social total score
COM, Communication total score
CDI, McArthur Communicative Development Inventory (Fenson et al., 1993)
RL, Receptive Language; EL, Expressive Language
CSBS DP, Communication and Symbolic Behavior Scales Developmental Profile (Wetherby and Prizant, 2002)
DAWBA, Development and Wellbeing Assessment (Goodman et al., 2000)
ESCS, Early Social Communication Scales (Mundy, 2003)
IBQ/ECBQ, Infant Behavior Questionnaire/Early Childhood Behavior Questionnaire (Gartstein and Rothbart, 2003)
MSEL, Mullen Scales of Early Learning (Mullen, 1995)
FM, Fine Motor; GM, Gross Motor; VR, Visual Reception; EL, Expressive Language, RL, Receptive Language
PLS, Preschool Language Scale (Zimmerman et al., 2009)
RL, Receptive Language; EL, Expressive Language
SCQ, Social Communication Questionnaire (Berument, Rutter, and Lord, 1999)
VABS, Vineland Adaptive Behavior Scales (Sparrow et al., 2005)
FM, Fine Motor; GM, Gross Motor; MS, Motor Skills; SOC, Socialization; COM, Communication; DLS, Daily Living Skills

RT, Reaction Time
*M*, Mean
*SD*, Standard Deviation *Additional references*: Berument, S., Rutter, M., Lord, C., 1999. Autism screening questionnaire: diagnostic validity. Br. J. Psychiatry 175, 444–451. Goodman, R., Ford, T., Richards, H., Gatward, R., Meltzer, H., 2000. The development and well-being assessment: description and initial validation of an integrated assessment of child and adolescent psychopathology. J. Child Psychol. Psychiatry, 41, 645–655. Mundy, P., Delgado, C., Block, J., Venezia, M., Hogan, A., Seibert, J., 2003. Early Social Communication Scales. University of Miami. Wetherby, A., Prizant, B., 2002. Communication and Symbolic Behavior Scales Developmental Profile: First Normed Edition. Brookes, Baltimore. Zimmerman, I.L., Pond, R.E., Steiner, V.G., 2009. Preschool Language Scale IV. Pearson.

## Footnotes

1 Pivotal Response Treatment (Koegel et al., 1999) is an evidence-based manualized intervention for individuals with ASD, and is one of 10 comprehensive model programs for children with autism identified by the US National Research Council (2001).

2 Promoting First Relationships focuses on promoting positive characteristics in the parent–child interaction and strengthening families; the intervention has shown efficacy in improving child responsiveness and contingency in preschoolers with disabilities (Kelly et al., 2008).
